# Modeling of whole brain sleep electroencephalogram using deep oscillatory neural network

**DOI:** 10.3389/fninf.2025.1513374

**Published:** 2025-05-14

**Authors:** Sayan Ghosh, Dipayan Biswas, N. R. Rohan, Sujith Vijayan, V. Srinivasa Chakravarthy

**Affiliations:** ^1^Indian Institute of Technology Madras, Chennai, India; ^2^Virginia Tech, Blacksburg, VA, United States

**Keywords:** EEG, Hopf oscillator, sleep stages modeling, large scale brain dynamics, biomedical signal analysis, Hopf oscillator model

## Abstract

This study presents a general trainable network of Hopf oscillators to model high-dimensional electroencephalogram (EEG) signals across different sleep stages. The proposed architecture consists of two main components: a layer of interconnected oscillators and a complex-valued feed-forward network designed with and without a hidden layer. Incorporating a hidden layer in the feed-forward network leads to lower reconstruction errors than the simpler version without it. Our model reconstructs EEG signals across all five sleep stages and predicts the subsequent 5 s of EEG activity. The predicted data closely aligns with the empirical EEG regarding mean absolute error, power spectral similarity, and complexity measures. We propose three models, each representing a stage of increasing complexity from initial training to architectures with and without hidden layers. In these models, the oscillators initially lack spatial localization. However, we introduce spatial constraints in the final two models by superimposing spherical shells and rectangular geometries onto the oscillator network. Overall, the proposed model represents a step toward constructing a large-scale, biologically inspired model of brain dynamics.

## 1 Introduction

Using extracranial electrodes, EEG measures the extracellular ionic current produced by a graded postsynaptic potential of vertically oriented pyramidal neurons in the III, V, and VI cortical layers (Nunez and Srinivasan, 2006). The electrical dipole field created by the soma and apical dendrites of pyramidal neurons is propagated through layers of the cortex, cerebrospinal fluid (CSF), skull, and scalp via volume conduction (Schaul, 1998) and recordable at the scalp site (Mule et al., 2021). EEG technology has diverse applications, including characterizing brain dynamics in the early stages of Parkinson’s disease (PD) (Han et al., 2013), epileptic seizure detection (Zhou et al., 2018), motor imagery and movement classification in brain-computer interfaces (Thomas et al., 2009), emotion classification (Alhagry et al., 2017) etc.

Oscillations are a key feature of sleep EEG and are crucial in various physiological and cognitive processes (Lambert and Peter-Derex, 2023). For example, the presence of theta waves and reduction of an alpha wave during stage 1 sleep, Stage 2 sleep is characterized by bursts of oscillatory activity and increased cortical synchrony; delta wave is a marker of deep sleep also affected by diseases like insomnia (Bakker et al., 2023), theta oscillation in the hippocampus during REM stage, etc. EEG-based Coherence (Watanabe et al., 2023) and phase synchronizations (Ojha and Panda, 2024) are the quantitative measures of functional connectivity during sleep and temporal coordination of neuronal activity. Achermann and Borbély (1997) shows the significance of neuronal synchrony during deep sleep and the increase in delta (0.1–4 Hz) and theta (4–7 Hz) band power following sleep onset, particularly in the fronto-central region. Studies also report the earlier emergence of alpha (8–12 Hz) and frontal theta oscillations after sleep deprivation (Gorgoni et al., 2019), the effect of transcranial oscillatory stimulation on memory consolidation during NREM sleep (Marshall et al., 2006), and the role of oscillations as biomarkers of sleep homeostasis such as theta activity during wakefulness and slow-wave activity (delta) during sleep (Finelli et al., 2000). Sleep also plays a major role in synaptic homeostasis and memory consolidation, emphasizing the importance of oscillatory activity in brain function (Esser et al., 2007; Pankka et al., 2024; Bahador et al., 2021; Paul, 2020; Sano et al., 2018; Michielli et al., 2019; Zhao et al., 2024; Burke and de Paor, 2004; Weigenand et al., 2014; Ospeck, 2019; Ghosh et al., 2025; Krishnamurthy et al., 2022; Buzsáki, 2006; Achermann et al., 1993). The EEG-based sleep transition model (Ising model) describes the synchronous dynamics of the neuronal population (Acconito et al., 2023). The Kuramoto model describes phase synchronization among different brain regions during sleep (Ingendoh et al., 2023).

In the past decade, several efforts have been made to model the brain as a nonlinear dynamical system and describe brain dynamics using complex nonlinear dynamical networks (Başar, 1983). A phenomenological model comprising van der Pol–Duffing double oscillator networks was used to model EEG signals from healthy controls as well as Alzheimer’s disease patients (Ghorbanian et al., 2015b). The model results compare favorably with experimental results in terms of time series, power spectrum, and Shannon entropy (Ghorbanian et al., 2015a; Ghorbanian et al., 2015b; Ghorbanian et al., 2014). Another model used coupled Duffing-van der pol oscillators to generate EEG ictal patterns from the temporal lobe (Szuflitowska and Orlowski, 2021). By analyzing EEG time series, the presence of low dimensional chaos in NREM N1 and REM sleep stages was described by Babloyantz et al. (1985). Studies have been made using stochastic limit cycle oscillators to model EEG data from healthy subjects (Rankine et al., 2006; Burke and de Paor, 2004).

Hopf oscillator networks offer a robust framework for modeling complex brain dynamics across different states (Burke and de Paor, 2004). Neural mass models have been used to explain slow-wave activity and K-complexes (Weigenand et al., 2014). Additionally, Hopf oscillators provide a powerful approach to modeling the oscillatory neural dynamics underlying memory consolidation and sleep spindles (Ospeck, 2019). Their properties closely align with observed sleep and memory research phenomena, offering insights into how the brain processes, stores, and consolidates information through oscillatory activity patterns. These models connect the neuronal-level dynamics and population-level behavior observed in EEG recordings.

Despite these efforts, the link between the mesoscopic EEG activity and the dynamics underlying neuronal circuits still needs to be fully unraveled. A weighted mean potential of a weakly-coupled, local cluster of Hindmarsh-Rose (HR) neurons collectively shows near-synchronization behavior and can optimally reconstruct epileptic EEG time series (Ren et al., 2017). A similar line of work was also proposed by Phuong and colleagues, in which networks of HR neurons and Kuramato oscillators were used to reconstruct EEG data in healthy and epileptic conditions (Nguyen et al., 2020). Ibáñez-Molina and Iglesias-Parro (2016) show a mean field of the Kuramoto oscillator to explore the dynamics of electroencephalographic (EEG) complexity during mind-wandering episodes. This work contributes to understanding how neural mechanisms underpin spontaneous thought processes and their representation in EEG signals. In another study (Das and Puthankattil, 2022), the authors used a phenomenological computational model of the Kuramoto oscillator to investigate functional connectivity and EEG complexity in mild cognitive impairment (MCI), a precursor to Alzheimer’s disease (AD). The study revealed that the brain’s dynamic repertoire results from the interplay between network topology and oscillatory dynamics by combining empirical structural and functional connectivity data with computational models (Kuramoto oscillatory model) of coupled oscillators (Cabral et al., 2022). This research highlights the role of synchronization mechanisms in shaping large-scale brain dynamics and offers a framework for understanding how the connectome supports diverse neural functions. Similar approaches have also been taken to model Functional magnetic resonance imaging (fMRI) (Logothetis et al., 2001) signals using the non-linear oscillators (Kuramoto, Hopf) model (Cabral et al., 2023; Breakspear, 2017; Deco et al., 2017). Several Hopf oscillatory models have been developed that explain several physiological phenomena such as cognitive behavior, sleep–wake cycle, Schizophrenia, and Alzheimer’s disease (Deco et al., 2015; Deco et al., 2017; Deco et al., 2021; Luppi et al., 2022; López-González et al., 2021; Deco and Kringelbach, 2014).

There is a growing interest in modeling large-scale brain activity using networks of nonlinear oscillators. A notable example of this kind is The Virtual Brain (TVB) framework, which uses large oscillatory networks to model various manifestations of functional brain dynamics like EEG, functional Magnetic Resonance Imaging (fMRI), and Magnetoencephalogram (MEG) (Sanz Leon et al., 2013). Al-Hossenat et al. (2019) proposed modeling of slow wave activity (delta and theta) of EEG from eight different regions using Jansen and Rit’s (JR) neural mass model and anatomical connectivity using the TVB framework. In another modeling study, using a similar kind of neural mass model, alpha wave activity was reproduced from four different brain regions (Al-Hossenat et al., 2017).

Several deep learning models have been proposed for EEG time series forecasting, including WaveNet (Pankka et al., 2024), correlation-based approaches (Bahador et al., 2021), and LSTM-based networks (Paul, 2020). LSTM-RNN networks have been applied to detect sleep stages from EEG signals (Sano et al., 2018; Michielli et al., 2019) and for sleep EEG reconstruction (Zhao et al., 2024), leveraging their ability to capture temporal dependencies in sleep EEG data. However, these networks primarily work in time series, not capturing the frequency and phase information. These approaches overlook key biological oscillatory features such as phase, frequency, and amplitude.

Feedforward spiking neural network models (Singanamalla and Lin, 2021; Zenke and Ganguli, 2018) generate synthetic EEG signals for Motor imagery and SSVEP EEG data. Yang et al. (2024) introduced a biologically inspired unsupervised learning framework for spiking neural networks (SNNs), enhancing neuromorphic vision systems with robust, efficient, and energy-adaptive visual perception for embodied applications in robotics and AI. The same group advanced SNN capabilities with a surrogate gradient learning framework (Yang and Chen, 2023a), demonstrating superior temporal precision and efficiency for neuromorphic computing and AI. Similar authors proposed SNIB (Yang and Chen, 2023b; Yang and Chen, 2024), a groundbreaking framework applying the nonlinear information bottleneck (NIB) principle to optimize the trade-off between information compression and retention, enabling more efficient, robust, and adaptive spike-based learning. There is a model of linearly coupled Hopf oscillatory model that can explain complex dynamics, and information processing of the human brain (Deco et al., 2017), where model dynamics depend on coupling coefficients among oscillators and oscillator amplitude (*μ*) (Deco et al., 2017). However, these kinds of models cannot accurately predict the neuroimaging data like fMRI (Hahn et al., 2021; Iravani et al., 2021).

Compared with fMRI, a smaller number of brain modeling approaches have been explored with EEG. EEG is less expensive than fMRI, more easily available, portable, and has high temporal resolution. Therefore, we can use all the advantages of EEG to understand brain dynamics. With this motivation, we propose a network of Hopf oscillators described in the complex domain and show how the network can be trained to model high-dimensional EEG data in the waking and sleep stages and publicly available BONN epilepsy dataset (Andrzejak et al., 2001). Sleep is a complex, naturally recurring dynamic process that occurs periodically in most animals (Březinová, 1974). Three critical physiological mechanisms or rhythms regulate sleep, viz., circadian rhythm, homeostasis, and ultradian rhythm (Fisher et al., 2013; Achermann and Borbély, 2003). Polysomnogram (Wolpert, 1969), which jointly measures brain electrical activity (EEG), muscle activity (Electromyogram—EMG), eye movement (Electrooculogram—EOG), and heart rate (Electrocardiogram—ECG), is a standard method of recording sleep activity. Sleep stages can be broadly categorized into five stages: waking, non-rapid eye movement N1 (NREM N1), non-rapid eye movement N2 (NREM N2), non-rapid eye movement N3 (NREM N3), and rapid eye movement (REM) sleep (Šušmáková, 2004). Sleep facilitates important neural and physiological functions, including memory consolidation (Siegel, 2001), and emotion control (Goldstein and Walker, 2014).

In earlier work, we showed how to achieve a stable phase relationship between oscillators with arbitrarily different frequencies using a special form of coupling known as power coupling (Biswas et al., 2021). The difficulties that arise in a pair of coupled oscillators, depicted by Arnold tongues, seem to be overcome effectively with power coupling. It was shown how networks of such coupled oscillator systems can be trained to learn a small number of EEG channels. In the present study, we add a hidden layer of sigmoidal neurons and geometrically constrain the network to accurately learn high-dimensional, “whole brain” EEG signals under various sleep conditions.

In this work, we have developed a Deep Oscillatory Neural Network (DONN) to reconstruct and predict sleep EEG and epileptic EEG time series. This type of model combines oscillatory neurons and sigmoid neurons (Ghosh et al., 2025). RNNs (Krishnamurthy et al., 2022) with gating mechanisms are excellent at sequence processing capabilities but fail to show biological plausibility. In contrast, neural activity in the brain exhibits complex dynamics characterized by key frequency bands such as alpha, beta, gamma, and theta (Buzsáki, 2006). Our proposed oscillatory network consists of Hopf oscillators, where each unit exhibits both amplitude and phase dynamics. The oscillators presented in the network effectively carry out a Fourier decomposition of the teaching signal (Biswas et al., 2021). Additionally, we demonstrate that a single oscillatory neuron is computationally more efficient than single LSTM neurons in terms of processing time, moreover, the DONN network has significantly fewer trainable parameters compared to an LSTM (Ghosh et al., 2025).

**Figure 1:**
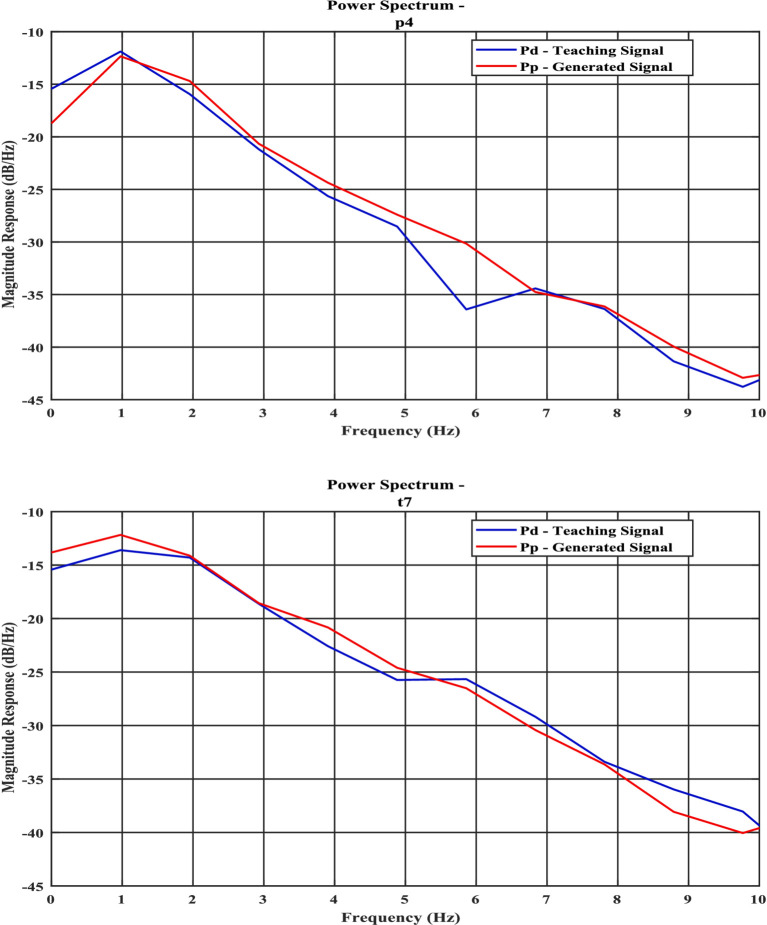
Power spectral density curves of experimental signal and model predicted signal for **(a)** NREM N1 for P4 channel; **(b)** NREM N3—“T7” channel, the blue line shows power spectrum of actual EEG Data (Pd) and orange line (Pp) shows model predicted power spectrum.

Our study shows that the EEG signal can be reconstructed as well as predicted optimally compared to the Kuramoto and HR neuron-based model proposed (Nguyen et al., 2020). Our model can reconstruct and predict the next 5 s of EEG data (2,500 data points) from 5 sleep stages. Various signal features like power spectrum density, Hurst exponent (Supplementary section 11), and Higuchi fractal (Supplementary section 12) dimension show good agreement with model-predicted EEG data compared to empirical EEG Data. The key contributions of this work are summarized below (1–5):

1. Modeling of different stages of sleep data. Reconstruction as well as prediction of future EEG data.
2. Statistical tests and error bar comparisons have been conducted with the existing literature. That shows significant improvement in contrast with the existing literature.
3. The predicted model signal exhibits significant similarity to real-time EEG data across different sleep stages, as evidenced by its power spectral density and Hurst component characteristics (Figure 1, Table 1; Supplementary sections 11–13).
4. We have created a spherical shell oscillatory model of the whole brain, where oscillators are spatially localized, which is a stepping stone toward large-scale brain modeling.
5. Find out optimal model parameters [Oscillator amplitude (*μ*), Coupling coefficient ( $\;\xi_{w}$ ), Beta (*β*) Additional Hidden Layer] (Section 3.4).

**Table 1:** Power spectrum mean error between model predicted and empirical EEG.

Sleep stages	Mean power spectrum error with std (%)
Wake	2.41 ± 0.48
NREM N1	3.35 ± 1.08
NREM N2	3.13 ± 1.16
NREM N3	3.92 ± 1.14
REM	3.51 ± 1.17

The results were obtained by averaging over 56 channels for each sleep stage.

The outline of the paper is as follows. This article begins with an account of sleep EEG recording followed by preprocessing methodology. The two stages of training of the proposed network are described in the ‘1st stage of training’ and ‘2nd stage of training’ sections, respectively. The section ‘Insertion of hidden layer’ describes the deep oscillator network, which is a combination of an oscillatory layer and a feedforward network. The section ‘Prediction of EEG Data’ describes the prediction of EEG data using a trained Hopf oscillator model. The section ‘spatial distribution of oscillator’ shows how oscillators are distributed on a rectangular grid and spherical shell geometry. Results from the reconstruction, EEG data prediction, and statistical analysis are described in the results section. A discussion of the work is presented in the last section.

## 2 Materials and methods

### 2.1 EEG recording

The Polysomnogram (PSG) datasets comprising 56 EEG, two electro-oculogram (EoG) and four EMG electrodes were recorded from two healthy subjects (full night, 8 h) at the School of Neuroscience, Virginia Tech, USA. During data collection, all necessary instructions, such as those regarding caffeine and alcohol use restrictions, were adhered to.

The different stages of sleep are scored according to the American Academy of Sleep Medicine (AASM) rules by two sleep experts (Singh et al., 2019). A night’s sleep consists of periods of rapid eye movement (REM) sleep and periods of non-rapid eye movement (NREM) sleep; the latter consists of three stages, NREM N1, NREM N2, and NREM N3, also known as slow-wave sleep. NREM N1 is often when a transition occurs between waking and sleep; the awake state is characterized by low amplitude and relatively high-frequency waves. NREM N1 occurs for 3%–8% of total full night sleep duration and is dominated by theta waves (4–7 Hz) (Březinová, 1974). NREM N2 is defined by sleep spindles (11–16 Hz) and K-complexes. NREM N3 is also called slow-wave sleep, as there is a prominent activity of the delta band (0.1 to 4 Hz). REM sleep is somewhat similar to the wake stage, which occurs more frequently late at night and occupies 20% of total sleep (Fisher et al., 2013). REM sleep is marked by muscle atonia and conjugate eye movements.

These datasets are in European Data Format (.EDF) format. For further analysis, we converted these datasets into .mat format using the EEGLAB (Iversen and Makeig, 2019) plugin in Matlab and extracted 10 s (training) and subsequent 5-s chunks (testing) from each of the 56 channels of EEG data. EEG is inherently a noisy signal influenced by non-neural factors [e.g., muscle movement measured by Electromyogram (EMG), eye movement measured by Electrooculogram (EOG), and Electrocardiogram (ECG)], as well as equipment noise (power line interference (50/60 Hz)), impedance fluctuation and, cable movements. In addition, EEG data is normalized to remove DC noise. The sampling frequency of the system is 500 Hz.

### 2.2 A network of neural oscillators

For our current purpose of modelling multi-dimensional EEG signals, we use an enhanced version of a network of neural oscillators described in Biswas et al. (2021). The original model of Biswas et al. (2021) consists of a layer of Hopf oscillators with lateral coupling connections and an output layer that is directly connected by a single linear weight stage to the oscillator layer. The dynamics of the Hopf oscillators were described in the complex domain, coupled using a unique form of coupling known as power coupling. The layer of oscillators is connected to the output layer using all-to-all linear forward weights. Thus, the given time series is modelled as a linear sum of the outputs of the layer of oscillators.

In the present model, a hidden layer of sigmoid neurons is inserted between the oscillatory layer and the output, immensely reducing the fitting error. The original network of Biswas et al. (2021) has two components: the input oscillatory layer consisting of a network of coupled Hopf oscillators and a feedforward linear weight stage that maps the oscillator’s outputs onto the network’s output node(s). We use the Hopf oscillator in the supercritical regime where the oscillator exhibits a stable limit cycle. In the previous study, we introduced ‘power coupling’, which shows how to achieve a constant normalized phase difference between a pair of coupled Hopf oscillators with arbitrary intrinsic frequencies (Biswas et al., 2021). The dynamics of the oscillatory layer are described by Equations 1a–1e.

### 2.3 The network of Hopf oscillators

The complex domain representation of a single Hopf oscillator is described as:


(1a) $$\dot{z}=z(\mu +\mathrm{i\omega}-|z^{2}|)$$

where z is a state variable,


(1b) $$z=re^{\mathrm{i\theta}},i=\sqrt{-1}$$

The dynamics of N-coupled Hopf oscillators without external input can be described as:


(1c) $$\dot{Z_{i}}=(\mu +i\omega_{i}+\beta |Z_{i}|2)Z_{i}+\sum_{j=1,∋j\neq i}^{N}A_{\mathrm{ij}}e^{i\frac{\emptyset_{\mathrm{ij}}}{\omega_{j}}}{Z_{j}}^{\frac{\omega_{i}}{\omega_{j}}}$$

The polar coordinate representation of Equation 1c is:


(1d) $$\dot{r_{i}}=(\mu +{{\mathrm{\beta r}}_{i}}^{2})r_{i}+\sum_{j=1\;∋j\neq i}^{N}A_{\mathrm{ij}}r_{j}^{\frac{\omega_{i}}{\omega_{j}}}\cos\;\omega_{i}(\frac{\theta_{j}}{\omega_{j}}-\frac{\theta_{i}}{\omega_{i}}+\frac{{Ø}_{\mathrm{ij}}}{\omega_{i}\omega_{j}})$$

(1e) $${\dot{\theta }}_{i}=\omega_{i}+\sum_{j=1\;∋j\neq i}^{N}A_{\mathrm{ij}}\frac{r_{j}^{\frac{\omega_{i}}{\omega_{j}}}}{r_{i}}\;\sin\;\omega_{i}(\frac{\theta_{j}}{\omega_{j}}-\frac{\theta_{i}}{\omega_{i}}+\frac{{Ø}_{\mathrm{ij}}}{\omega_{i}\omega_{j}})\;$$

where r and θ are the state variables, $\left(\sqrt{\frac{\mu }{\beta }}\right)$ is the amplitude of oscillation, μ and *β* are bifurcation parameter. In this brief *μ* = 1, *β* = −20. $A_{\mathrm{ij}}$ , is the magnitude of the complex coupling coefficient, ( $A_{\mathrm{ij}}\ll 1$ ), $\;{Ø}_{\mathrm{ij}}$ is the angle of the complex coupling coefficient, $\;\theta_{i}\;$ and $\omega_{i}$ and are the *i*^th^ oscillator’s phase and intrinsic frequency, respectively.

The network described above is trained in two phases: in stage 1, the intrinsic frequencies, $\omega_{i}$ , of the oscillators and the coupling weights among the oscillators ( $W_{\mathrm{ij}}^{\prime }$ ) in the oscillatory layer are trained; in stage 2, the feedforward linear weights between the oscillatory layer and the network output are trained.

### 2.4 1st stage of training

**Figure 2:**
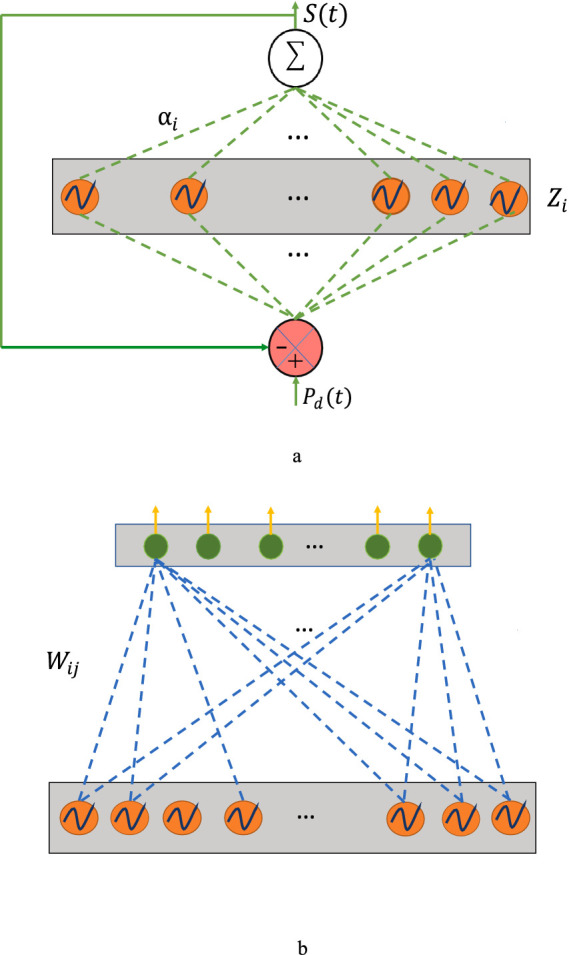
**(a)** Network architecture: 1st stage of training. **(b)** Network of 2nd stage of training.

Since the aim of the 1st stage of training (Figure 2a) is to train the intrinsic frequencies, $\omega_{j}$ , these frequencies are initialized by sampling from a uniform random distribution over the interval [0, 10] Hz. The modified network dynamics is described in Equation 2a, where the error signal e(t) drives each oscillator. The teaching signal used for training is denoted by, D(t) , which is an EEG signal of a finite duration. The power coupling weight, $W_{\mathrm{ij}}$ , in Equation 2b, is the complex lateral connection, ${Ø}_{\mathrm{ij}}$ is the angle of lateral connection and $A_{\mathrm{ij}}$ is the magnitude of the lateral connection between the $i^{\mathrm{th}}$ and $j^{\mathrm{th}}$ oscillators, and $\omega_{i}$ , is the intrinsic frequency of the i’th oscillator. The oscillator activations are summed using feedforward weights, $\alpha_{i}$ , which, in this stage of training, are taken to be small real numbers 0.2 (i.e., $\alpha_{i}$ =0.2 for all i) and the lateral connection $W_{\mathrm{ij}}^{\prime }$ is initialized with complex numbers according to Equation 2b. The training of intrinsic frequency, $\omega_{i}$ , is described in Equation 2c, where $\eta_{\omega }$ is the learning rate, e(t) is the error signal, and $\theta_{j}\;$ is the oscillator phase. The whole network is driven by an error signal, e(t), which is the difference between the network’s predicted signal and the desired signal Equation 2d. Training of the real feedforward weights is done by modified delta rule, Equation 2e, where $\eta_{\alpha }$ is the learning rate of feed-forward weight update. s(t) is the network reconstructed signal shown in Equation 2f. The network performs a Fourier-like decomposition of the target signal, and each oscillator is used to learn the frequency component closest to its own intrinsic frequency present in the signal. In the 1st stage, the intrinsic frequency, the angle of power coupling weight, and the amplitude of the signal (real feedforward weight) are trained. Lateral connections are trained by a complex-valued Hebbian rule (Equation 2g).


(2a) $$\dot{Z_{l}}=(\mu +i\omega_{i}+\beta |Z_{i}|2)Z_{i}+\sum_{j=1,∋j\neq i}^{N}A_{\mathrm{ij}}e^{i\frac{{Ø}_{\mathrm{ij}}}{\omega_{j}}}{Z_{j}}^{\frac{\omega_{i}}{\omega_{j}}}+\mathrm{\varepsilon e}(t)$$

The weight of power coupling can be written as Equation 2b:


(2b) $$W_{\mathrm{ij}}^{\prime }=\xi_{w}*A_{\mathrm{ij}}*e^{i\frac{{Ø}_{\mathrm{ij}}}{w_{j}}}$$

Frequency adaptation is done by the following rule:


(2c) $$\dot{\omega_{j}}=-\eta_{\omega }e(t)\sin\;\theta_{j}$$

(2d) e(t)=(D(t)−s(t))

Where D(t) is the teaching EEG time series and *s*(*t*) is the network predicted time series and *e*(*t*) is the output error.


(2e) $$\dot{\alpha_{l}}=\eta_{\alpha }(D(t)-s(t))\text{coscos}\;\theta_{j}\;$$

(2f) $$s(t)=\sum_{i=1}^{N}\alpha_{i}\cos\theta_{i}$$

Hebbian learning of complex power coupling is shown in Equation 2g


(2g) $$\tau_{w}\dot{W_{\mathrm{ij}}^{\prime }}=-W_{\mathrm{ij}}^{\prime }+z_{i}{\left(z_{j}^{*}\right)}^{\frac{\omega_{i}}{\omega_{j}}}$$

where $\tau_{w}$ is the time constant.

### 2.5 2nd stage of training

In the 2nd stage of training (Figure 2b), the oscillatory network with learned intrinsic frequencies and lateral connections of the oscillatory layer from the previous stage was used as a starting point. [Note that the oscillatory layer with trained parameters may be compared to a reservoir of *reservoir computing* (Biswas et al., 2021)].

But the main difference is that, in this stage, the feedforward weights are allowed to be complex (they were real in 1st stage training) and trained once again by supervised batch mode learning rule. $K_{\mathrm{ij}}$ and $\xi_{\mathrm{ij}}$ are the magnitude and angle of complex feedforward weights updated according to Equations 3c, 3d.

The complex feedforward weights are trained as follows (Equations 3a, 3b):


(3a) $$W_{\mathrm{ij}}^{\prime }=K_{\mathrm{ij}}e^{i\xi_{\mathrm{ij}}}\;$$

where $K_{\mathrm{ij}}$ and $\xi_{\mathrm{ij}}$ are the magnitude and angle of the complex feed-forward weight.


(3b) $$Y_{\mathrm{pi}}(t)=\text{real}(\sum_{j=1}^{n}W_{\mathrm{ij}}^{\prime }e^{i\theta_{j}})\;$$

The update rules for $K_{\mathrm{ij}}$ and $\xi_{\mathrm{ij}}$ are discussed in Equations 3c, 3d.


(3c) $$\Delta K_{\mathrm{ij}}=\eta_{K_{\mathrm{ij}}}\frac{\partial L}{\partial K_{\mathrm{ij}}}=(-1)\eta_{k}\sum_{t}(Y_{\mathrm{di}}(t)-Y_{\mathrm{pi}}(t))\cos(\theta_{j}(t)+\xi_{\mathrm{ij}})$$

(3d) $$\begin{array}{l} \Delta \xi_{\mathrm{ij}}=\eta_{\emptyset_{\mathrm{ij}}}\frac{\partial L}{\partial \emptyset_{\mathrm{ij}}}=(-1)\eta_{Ø}\sum_{t}(Y_{\mathrm{di}}(t)-Y_{\mathrm{pi}}(t))\\ \;(-K_{\mathrm{ij}}\sin\;(\theta_{j}(t)+\xi_{\mathrm{ij}})\;)\;\; \end{array}$$

The number of epochs for complex feedforward weight learning is 5,000, and learning parameters are $\eta_{k}=3\times 10^{-5}$ , $\eta_{Ø}$ = $10^{-6}$ .

### 2.6 Insertion of the hidden layer

**Figure 3:**
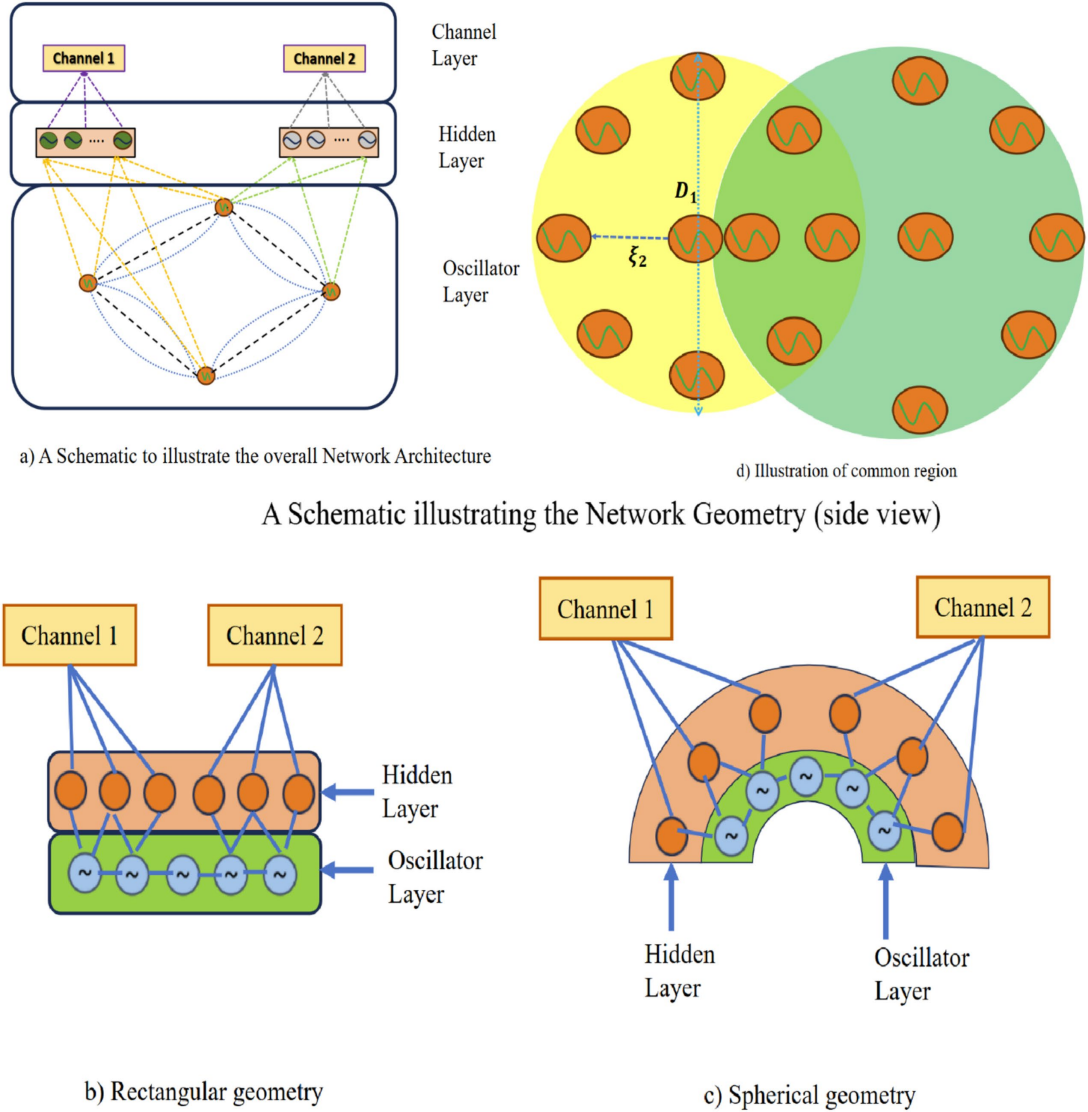
**(a)** A schematic to illustrate the overall network architecture. **(b,c)** A schematic illustrating the network geometry (side view). **(d)** A schematic illustration of shared oscillator’s common region between two channels (top view).

As we will see subsequently in the result section, despite the theoretical advantages, the model described above needs to yield satisfactory approximations of the empirical EEG signals. We insert a hidden layer of sigmoidal neurons between the oscillatory layer and the output to improve the approximation performance (Figure 3a). In the new version of the model with the hidden layer, the intrinsic frequencies of the oscillators and their lateral connections are trained using the learning mechanisms of the 1st stage of training described above (Equations 2a–2f). The mathematical derivation of hidden layers has been described in Supplementary section 3. Also, we have introduced two geometrical configurations: (a) rectangular and (b) spherical in this latest version of the model (Figures 3b,c), where a few oscillators are shared among EEG channels (Figure 3d).

### 2.7 Generation of EEG

So far, using supervised training, we have only reconstructed the data. To validate our model, we have to generate the output of the model without the network being driven by the training signal. The network can generate next the 5-s (2,500 samples) EEG signal without any external input. During generation, phases of oscillators, intrinsic frequency of oscillators, all feedforward weights (oscillatory layer to hidden layer weights and hidden layer to output layer weights) are adopted from the trained network. Here r and *ϕ* dynamics (Equations 4a, 4b) are derived by transform from complex variable representation (Equation 2a) to polar coordinates. r and *θ* dynamics are given below:


(4a) $$\dot{r_{i}}=(\mu +{{\mathrm{\beta r}}_{i}}^{2})r_{i}+\sum_{j=1\;∋j\neq i}^{N}A_{\mathrm{ij}}r_{j}^{\frac{\omega_{i}}{\omega_{j}}}\cos\;\omega_{i}(\frac{\theta_{j}}{\omega_{j}}-\frac{\theta_{i}}{\omega_{i}}+\frac{{Ø}_{\mathrm{ij}}}{\omega_{i}\omega_{j}})$$

(4b) $$\dot{\theta_{i}}=\omega_{i}+\sum_{j=1\;∋j\neq i}^{N}A_{\mathrm{ij}}\frac{r_{j}^{\frac{\omega_{i}}{\omega_{j}}}}{r_{i}}\;\sin\;\omega_{i}(\frac{\theta_{j}}{\omega_{j}}-\frac{\theta_{i}}{\omega_{i}}+\frac{{Ø}_{\mathrm{ij}}}{\omega_{i}\omega_{j}})$$

**Figure 4:**
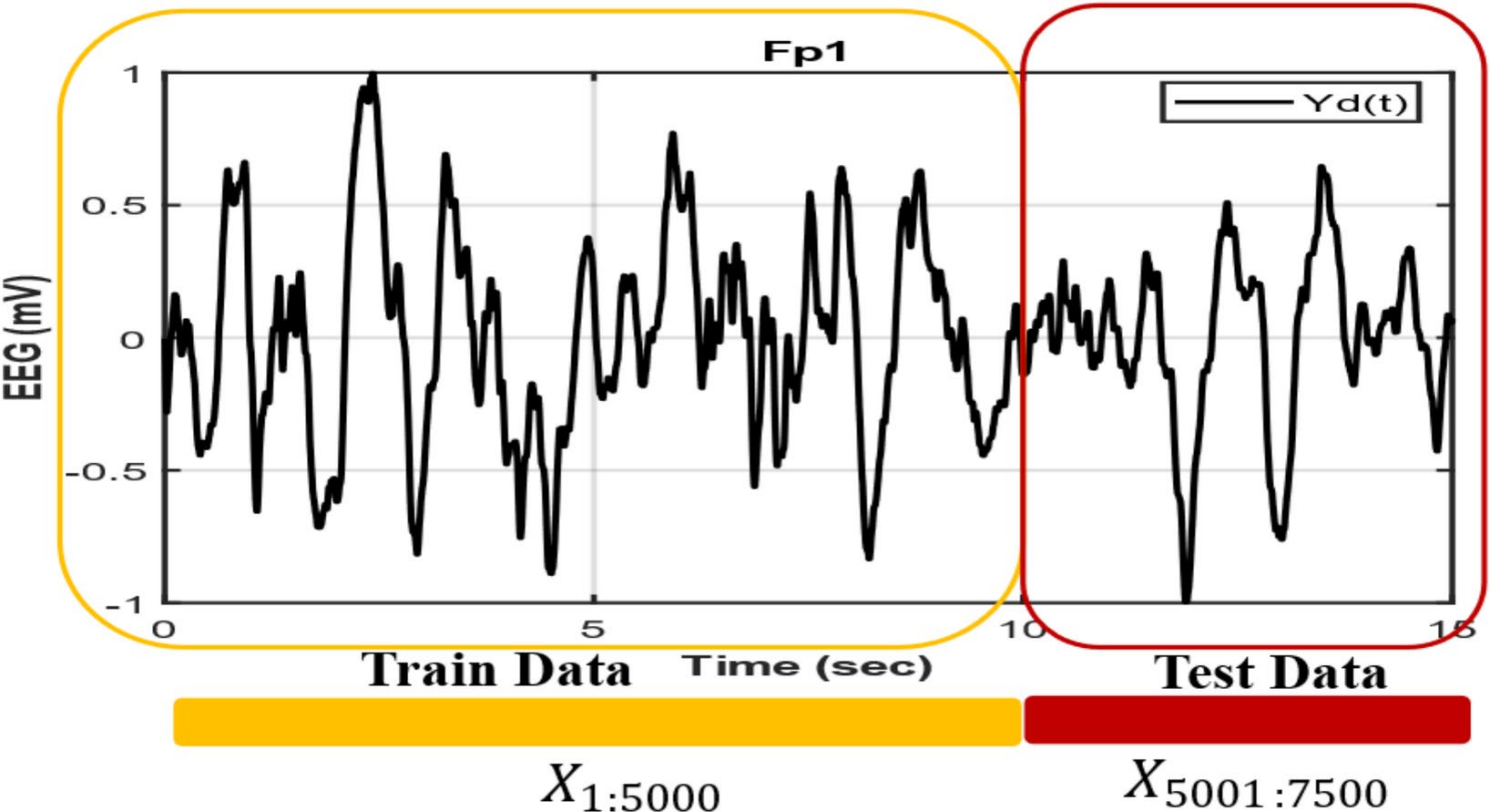
EEG signals are divided into training and testing segments, where an initial 10-s interval is used for training and the following 5-s segment is used for testing.

In Figure 4 we show how we construct the network and duration of the training and generating segment, where the first 5,000 points are used as training data taken from empirical EEG data, and last 2,500 points, which is not used during the training phase, are used for generation. Empirical EEG data has been divided for train and next 5 s EEG data is used to compare with the network generated output. To validate our model, we compare it with the method proposed in Nguyen et al. (2020) in which the Kuramoto oscillator and Hindmarsh-Rose (HR) neuron are used to produce EEG signal. Note that we extracted the train data in such a way that the next 5 s segment also belongs to the same sleep stage as that of the training segment (Figure 4).

### 2.8 Spatial distribution of oscillators

In the models described above, there is no spatial organized superimposed on the oscillators. In order to impart a greater realism and biological plausibility, we now impose a spatial organization on the proposed oscillator network. To this end, we consider two spatial distributions of the oscillators within a “cortical layer” which is modeled in two ways: (1) a spherical shell, and (2) a spherical shell a rectangular grid (Supplementary material). Electrodes are placed on the top of the cortical layer, inside another layer named the “electrode layer”. Real-world 10–20 electrode geometry is introduced in the “spherical shell” case.

Next, we specify which oscillators in the cortical layer contribute to which electrodes in the electrode layer. This is done by a simple nearest neighbor criterion: only the oscillators that lie within a threshold distance ( $\xi_{1}$ ) from a given electrode contribute to that electrode, as defined in Equation 5a.


(5a) $$D_{1}=\sqrt{{\left(x_{\mathrm{ch}}-x_{\mathrm{os}}\right)}^{2}+(y_{\mathrm{ch}}-y_{\mathrm{os}})+{\left(z_{\mathrm{ch}}-z_{\mathrm{os}}\right)}^{2}}$$

where $D_{1}$ is the distance from a given electrode in the electrode layer to a given oscillator in the cortical layer, and where $D_{1}$ is the distance from the electrode layer to the cortical layer. And $\left(x_{\mathrm{ch}},y_{\mathrm{ch}},z_{\mathrm{ch}}\right)$ and $\left(x_{\mathrm{os}},y_{\mathrm{os}},z_{\mathrm{os}}\right)$ is the Cartesian coordinate representation of electrode layer and cortical layer, respectively.

There is also another layer named “hidden layer” in between the cortical layer and the electrode layer. No specific spatial location is specified for the neurons in the hidden layer. Here basically the model architecture followed was similar to that described in Section 2.6, Figure 3a but with one important difference: a separate hidden layer of neurons is introduced for every electrode. In the schematic shown in Figures 3b,c, two different hidden layers are depicted corresponding to two distinct electrodes. The corresponding oscillators are also shown. Note that though the hidden layers are not shared between two electrodes, the corresponding oscillators can be partially shared (Figures 3b,c).

The thresholding process mentioned in above determines the connectivity between the electrodes and the oscillators. We use another threshold that determines the lateral connections among the oscillators. Here we use the spatial location of the oscillators in the cortical layer to specify local connectivity using another distance-based threshold ( $\xi_{2}$ ) derived in Equation 5b. Thus long-range connections among the oscillators are avoided.

Consider $D_{\mathrm{ij}}^{\prime }$ to be the distance between the $i^{\mathrm{th}}$ and the $j^{\mathrm{th}}$ oscillators. A pair of oscillators whose mutual distance exceeds the distance threshold limit $\;(\xi_{2}$ ), are not connected. $A_{\mathrm{ij}}^{\prime }$ represents the magnitude of the complex-valued connection, $W_{\mathrm{ij}}^{\prime }$ , between $i^{\mathrm{th}}$ and $j^{\mathrm{th}}$ oscillators; $A_{\mathrm{ij}}^{\prime }$ is set to 0.001. Only those oscillator pairs are connected and trained which are within the threshold distance ( $\xi_{2}$ ) of each other, i.e., $i^{\mathrm{th}}$ and $j^{\mathrm{th}}$ oscillator are connected only if $D\prime_{\mathrm{ij}}<\xi_{2}$ where,


(5b) $$D_{\mathrm{ij}}^{\prime }=\sqrt{{\left(x_{\mathrm{os},i}-x_{\mathrm{os},j}\right)}^{2}+{\left(y_{\mathrm{os},i}-y_{\mathrm{os},j}\right)}^{2}+{\left(z_{\mathrm{os},i}-z_{\mathrm{os},j}\right)}^{2}}$$

if $D\prime_{\mathrm{ij}}<\xi_{2}$


$$A_{\mathrm{ij}}^{\prime }=|W\prime_{\mathrm{ij}}|=0.001$$

else


$$A_{\mathrm{ij}}^{\prime }=|W\prime_{\mathrm{ij}}|=0$$

Where ( $x_{\mathrm{os},i},y_{\mathrm{os},i},z_{\mathrm{os},i}$ ) is the Cartesian coordinate representation of $i^{\mathrm{th}}$ oscillator and ( $x_{\mathrm{os},j},y_{\mathrm{os},j},z_{\mathrm{os},j}$ ) is the Cartesian coordinate representation of $j^{\mathrm{th}}$ oscillator.

Note that the angle of the coupling connections, $W\prime_{\mathrm{ij}}$ , is calculated by Hebbian learning as per Equation 2g.

### 2.9 Spatial distribution of the oscillators in a spherical shell

In this section, we describe a model in which the cortical layer is modeled as a spherical shell. Likewise, in reality, EEG electrodes also are not confined to a planar surface. We currently place the electrodes on a spherical surface on top of a spherical cortical layer. We extracted the precise electrode locations from EEGLab (Iversen and Makeig, 2019). Based on the position and radius of electrode layer we create a spherical shell. A set of 8 electrodes are shown on the cortical layer in Figure 3c. Similar to the case of the rectangular grid (Supplementary section 10), in this case too, there is a hidden layer between the cortical layer, which consists of oscillators, and the electrode layer. Network training is performed as per the equations described in Section 2.4 (Equations 2a–2g and hidden layer equation described in Supplementary section 3).

## 3 Results

In this section, we describe the performance of the models described in the previous section on modelling high-dimensional, whole-brain EEG data (56 electrodes). In order to model a large number of electrodes, as well as the essential frequency band (0.1 to 20 Hz), a large number (N = 200) of oscillators are used. Although the model is trained on the original EEG time series. In order to depict the signal spectrum, instead of using normal FFT, we use average periodogram method known as the “Welch method” (Naderi and Mahdavi-Nasab, 2010). We use the Welch method with a Hamming window of size 1 s and 50% overlap throughout the paper.

### 3.1 Reconstruction without hidden layer

#### 3.1.1 1st stage of training

Following the method described in Section 2.4, we show how the intrinsic frequency of the oscillators adapts to the nearby frequency components present in the desired signal. The intrinsic frequencies of the oscillators, $\omega_{i}$ , are initialized by drawing from a uniform distribution over the interval [0, 10] Hz. The real feedforward weight ( $\alpha_{i}$ ) which connects $i^{\mathrm{th}}$ oscillator to the single output node is uniformly initialized with a small real number (=0.2). The complex-valued lateral connections, $W_{\mathrm{ij}}^{\prime }$ , are initialized according to Equation 2b. Note that in this stage, we do not use the hidden layer in the feedforward stage. Training is performed for 30 epochs.

EEG chunks of duration 10 s are used for training. Frequency learning rate is $\eta_{w}=0.0001$ (Equation 2c), amplitude learning rate $\eta_{\alpha }$ = 0.0001 (Equation 2e), and the learning rate for the coefficient of lateral connection weight, which determines the magnitude of oscillator-to-oscillator connections ( A ), (Equation 2b), is $\;\xi_{w}\;$ = 0.001.

#### 3.1.2 2nd stage of training

Following the method described in Section 2.5, in this stage, we use the learned intrinsic frequencies and lateral connections from the 1st stage of training, while amplitude and phases of the complex feed-forward weight ( $W_{\mathrm{ij}}$ ) are trained further. Although the equation shows a single electrode signal, by using a matrix of feedforward weights we can reconstruct any number of channels. Note that learning rate for weight magnitude ( $\eta_{k}$ ) is 0.00003, and the learning rate for angle learning is $\eta_{Ø}$ is 0.000001; n is the number of channels to be reconstructed. Therefore, the predicted signals (Supplementary Figure 2) from the 2nd stage look better than the 1st stage (Supplementary Figure 1). Whereas in the 1st stage we use real feedforward weights, in the 2nd stage we use complex feedforward weights: this is the only difference between the two stages of training. The ‘power coupling’ rule for coupling the oscillators was developed to produce a constant normalized phase difference among the oscillators (Biswas et al., 2021). We compare the results obtained with power coupling, with other forms of coupling in Table 2.

**Table 2:** Comparison table of model performance with different power coupling methods.

Types of coupling	Trainable parameters	Model performances MAE error	
		Training	Generation
Linear coupling (all to all)	None	0.0474	10.62
Linear coupling (nearest neighbour)	None	0.0458	11.17
Complex coupling	The natural frequency of the oscillator, lateral connection, feedforward weights	0.0277	7.82
Power coupling (our proposed)	Natural frequency, Lateral connections, feedforward weights	0.01–0.02	5.56–6.67

### 3.2 Reconstruction with hidden layer (an alternative approach to 2nd stage training)

**Figure 5:**
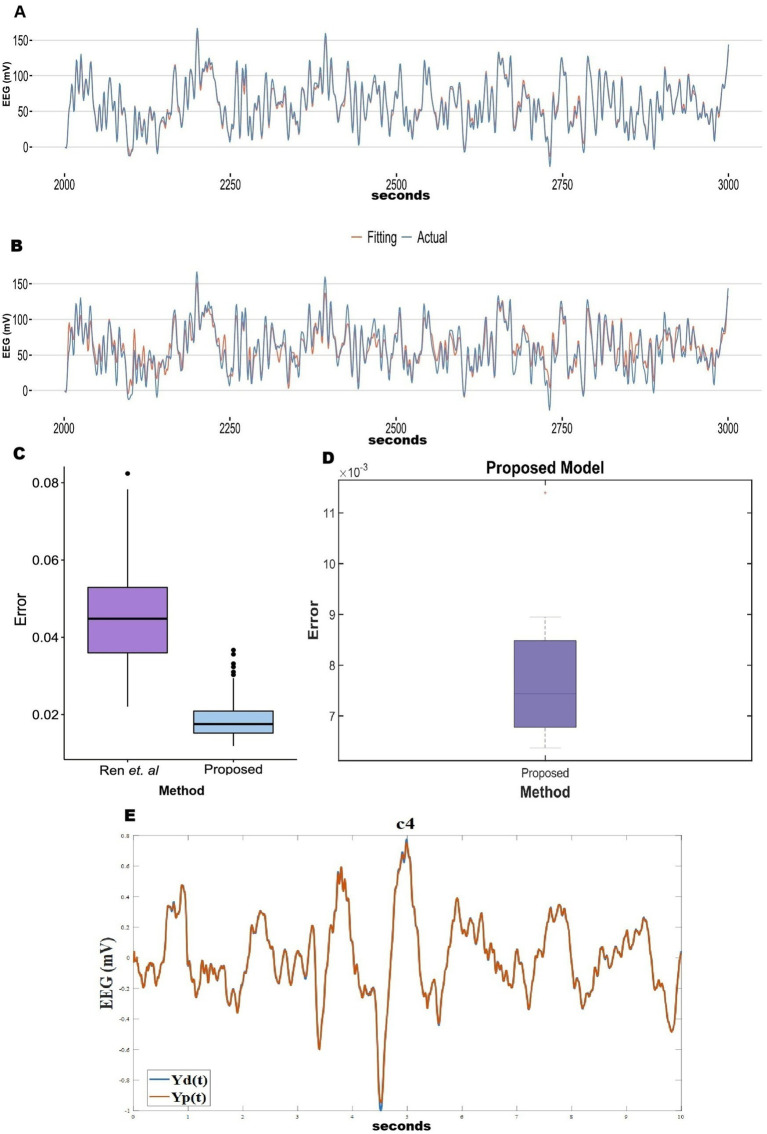
**(A–C)** Adopted from Nguyen et al. (2020). **(C)** Comparison between Ren et al. (2017) and Nguyen et al. (2020). **(D)** Reconstruction (during training) mean absolute Error distribution during training between EEG signals (our proposed method). **(E)** Blue line shows actual EEG and orange line show model reconstruction during training (Our proposed method).

In the previous section, we observed that the reconstruction error is poor when there is no hidden layer. To improve the model performance, following the method described in Section 2.6, we inserted a hidden layer of 100 sigmoidal neurons between the oscillatory layer and the output layer in the 2nd stage of training (learning rates: $\eta_{h}$ =0.001; $\eta_{o}$ =0.001). This modification greatly improved performance, with the RMSE values dropping by an order of magnitude. Comparisons between the cases of “without hidden layer” and “with hidden layer” model prediction for all the five stages of sleep are shown in a bar plot (Supplementary Figure 4). How reconstruction RMSE changes with the change in hidden layer neurons, has been shown in a bar plot (Supplementary Figure 5). Also, the generality of the network has been described in terms of functional connectivity analysis (Supplementary section 8). Mean absolute error (MAE) of the reconstruction of our model has been compared with Ren et al. (2017) and Nguyen et al. (2020) (Figure 5). Our proposed model has reconstruction error in the range of (0.007–0.019). In comparison with Ren et al. (2017) and Nguyen et al. (2020) time series fitting and training error bar plots are shown in Figures 5A–E. Figures 5A–C are adapted from Nguyen et al. (2020); licensed under CC BY 4.0. From Figures 5C,E we can conclude that our proposed method performs better than the other two methods (Ren et al., 2017; Nguyen et al., 2020).

### 3.3 Generation of EEG data using trained network

Here we generate EEG data for 5 s (after training with a 10 s-long EEG segment) using the trained parameters—intrinsic frequency of oscillators, lateral coupling weights among oscillators, and feed-forward weights [training of intrinsic frequencies and lateral are discussed in Section 2.5, feed-forward weight training rules are described in Section 2.6 (insertion of hidden layer)]. We have used MSE loss function in this model. The model parameters of this network has been described in Table 3.

**Table 3:** Model parameter of the “Insertion of hidden layer” model described in Figure 3a.

Network parameter	Size of the parameter
EEG electrodes	56
Number of Hopf Oscillators	250
Number of neurons in 1st hidden layer	100
Oscillator to 1st hidden layer weight	100*250
Type of neuron in 1st hidden layer	Tanh
Number of neuron in Output layer	56
1st hidden layer to output layer weight	56*100
Type of neuron in output layer	Tanh
Loss function	MSE
Epoch	2000

**Figure 6:**
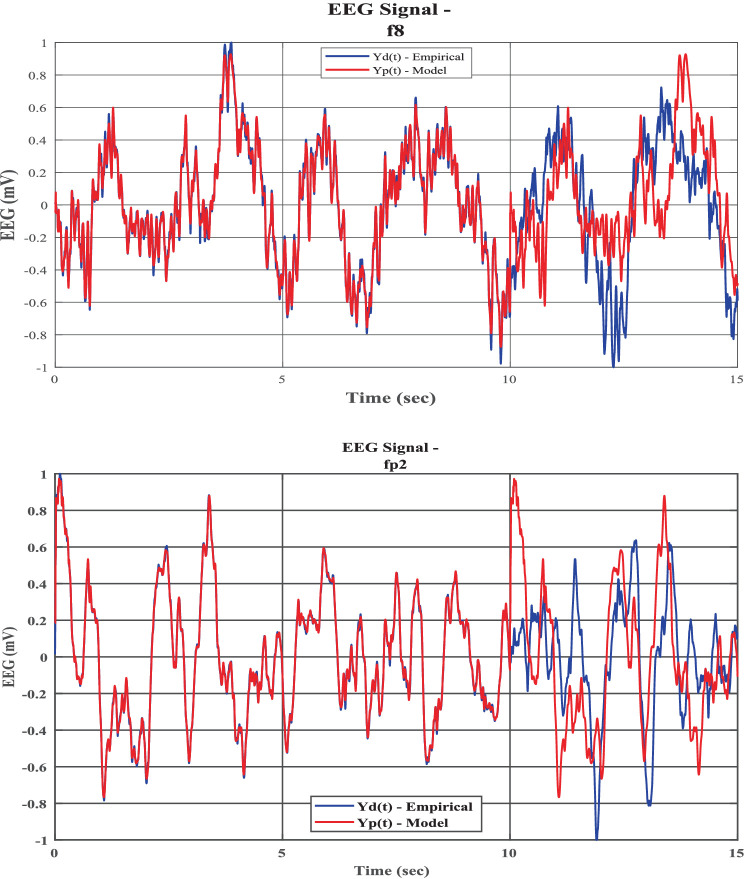
**(a,b)** Empirical EEG Data and training and generated (model reconstructed and model generated) of five sleep stages [**(a)** NREM N1-F8 channel; **(b)** NREM N3: Fp2 channel]. The blue line shows actual EEG Data (Yd(t)) and orange line (Yp(t)) shows model reconstructed (initial 10 s) and then generated output (next 5 s). Where initial 10 s data are reconstructed and 5 s data are generated from model.

The power spectrum (Figures 1a,b), time series (Figures 6a,b), Hurst exponent (HC) (Supplementary section 11), and Higuchi fractal dimension (HFD) (Supplementary section 12) of the predicted signal are compared with the next 5 s segment of empirical EEG data. The time series of the training segment as well as the generated segment are plotted (Figures 6a,b) for two sleep stages. (The other three sleep stages have been shown in the Supplementary Figures 7a–c).

To evaluate our model performance, we calculated mean absolute error (MAE) (Nguyen et al., 2020), which basically determines the time average difference between network simulated data (model prediction) and empirical EEG Data. MAE can be mathematically expressed as Equation 6a:


(6a) $$\mathrm{MAE}=\sum_{t1}^{t2}\frac{|Y_{p}-Y_{d}|}{\left(t_{2}-t_{1}\right)}$$

where $Y_{p}$ (predicted signal for a time interval $\left(t_{2}-t_{1}\right)$ ) is predicted EEG and $Y_{d}$ is the actual EEG, and their absolute difference is averaged over a time window.

The average error score (average over all channels for each sleep stage) was calculated using MAE (Equation 6a). The result of MAE values along with standard deviation are listed in Table 4.

**Table 4:** MAE prediction error (%) between model predicted and empirical EEG.

Sleep stages	Mean absolute error with std (%)
Wake	6.37 ± 0.8223
NREM N1	6.2505 ± 1.0399
NREM N2	5.3034 ± 0.6341
NREM N3	6.0781 ± 0.7996
REM	5.6575 ± 0.9300

The results were obtained by averaging over 56 channels for each sleep stage.

**Figure 7:**
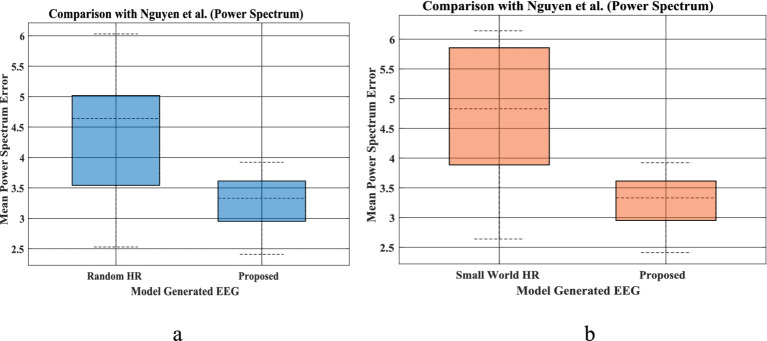
**(a,b)** EEG mean power spectrum during prediction proposed by Nguyen et al. (left bar in each figure), and our proposed coupled Hopf network-with hidden layer model (Figure 3A, Section 2.6) (right bar plot in each figure). The bar plot is calculated using power spectrum prediction error for all 5 set of EEGs [(i)-Set A, (ii)-Set B, (iii)-Set C, (iv)-Set D, (v)-Set E] for 4 different networks proposed by Nguyen et al. Similarly, power spectrum prediction calculated from our proposed model for all 5 sleep stages [(i)-Wake, (ii)-NREM N1, (iii)-NREM N2, (iv)-NREM N3, (v)-REM]. [**(a)** Comparison between Random HR and proposed; **(b)** comparison between small world HR and proposed].

Power spectrum density is one of the critical methods to quantify the EEG data. It resembles the frequency content of a signal. The spectral features of the generated EEG segments are compared over the test duration [the next 5 s after the training duration (Figures 1a,b)]. The empirical power is calculated from the actual EEG data and the predicted spectrum from the model predicted signal. The average power spectrum error over 56 channels for each sleep stage between model predicted and empirical EEG is shown in Table 1. Note that compared to Nguyen et al., our model power spectrum prediction error is significantly lesser (Figures 7a,b).

**Figure 8:**
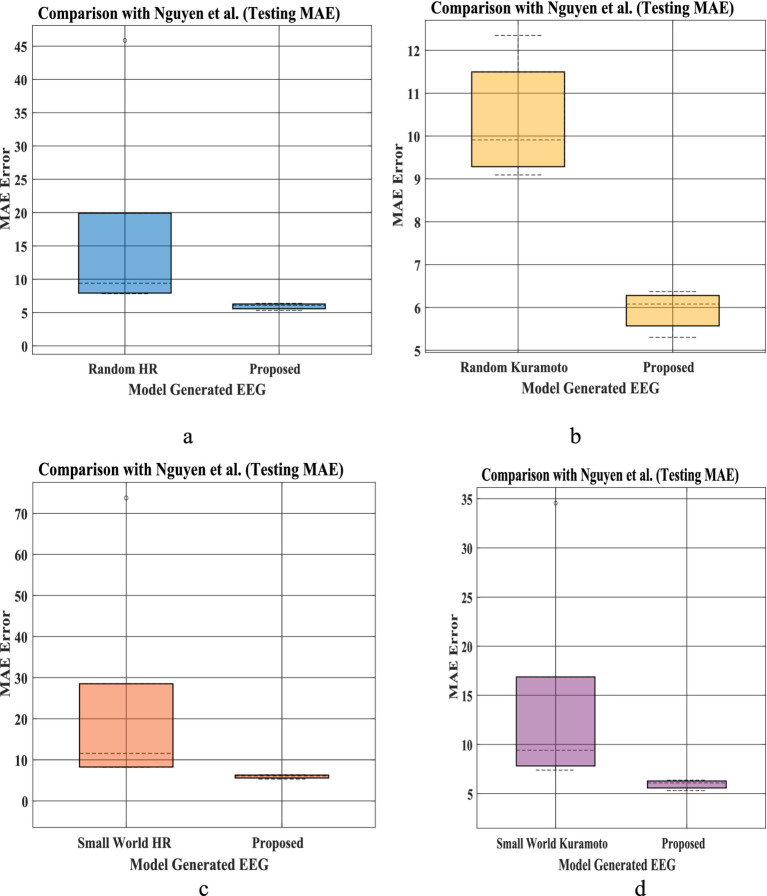
**(a–d)** EEG Mean absolute error (MAE) distribution for prediction proposed by Nguyen et al. (2020) (left bar in each figure for five set of EEG data), and our proposed with hidden layer model (Figure 3a, Section 2.6) (right bar plot in each figure). Bar plot is calculated using MAE of prediction for all 5 set of EEG [(i)-Set A, (ii)-Set B, (iii)-Set C, (iv)-Set D, (v)-Set E provided in the testing result section (Nguyen et al., 2020)] for 4 different network proposed by Nguyen et al. Similarly, MAE prediction calculated from our proposed model for all 5 sleep stages [(i)-Wake, (ii)-NREM N1, (iii)-NREM N2, (iv)-NREM N3, (v)-REM]. [**(a)** Comparison between Random HR and proposed; **(b)** comparison between small world HR and proposed; **(c)** comparison between Random Kuramoto and proposed; **(d)** comparison between small world Kuramoto model and the proposed model].

The bar plot in Figures 8a–d shows that mean absolute error between predicted and empirical EEG Data is less than the method proposed by Nguyen et al. To compare the mean error between our proposed network and that of Nguyen et al., we conduct Wilcoxon signed rank test. Our model produce a better fit [Test statistics (2) is less than critical value (8) for alpha value 0.05], indicating a significant difference between our proposed model and model proposed by Nagual et al. Also, Univariate statistical *t*-test was done to identify the relative significance of power spectral density between the EEG signal predicted by the model and empirical EEG Data (Supplementary section 13).

In this study, we also utilize an additional benchmark dataset from Nguyen et al. (2020) to evaluate the performance of our Hopf network. By comparing the MAE, power spectrum error, and Hurst component error metrics proposed in Nguyen et al. (2020), we demonstrate that our model achieves more promising results, outperforming the reference model. To compare this we have taken the BONN dataset (Andrzejak et al., 2001) and the details description of the BONN dataset has been explained into the Supplementary section 15. To maintain the consistency with the previous literature (Nguyen et al., 2020), here also we train with 1st 2,000 time points EEG and next 1,000 time points we used for testing (see Table 5).

**Table 5:** Comparison with Nguyen et al. (2020) and our proposed model on BONN dataset.

Data	Random HR	Small world HR	Random Kuramoto	Small world Kuramoto	Generative oscillatory neural network(proposed)
(a) MAE error					
Set A	11.28 ± 40.79	13.43 ± 58.88	12.35 ± 47.34	10.97 ± 40.05	7.34 ± 1.28
Set B	7.82 ± 15.90	11.58 ± 50.44	9.35 ± 20.26	7.95 ± 15.98	6.85 ± 0.74
Set C	7.95 ± 6.69	8.27 ± 9.11	9.09 ± 98.54	7.39 ± 10.49	5.71 ± 1.92
Set D	9.40 ± 10.50	8.17 ± 7.48	9.91 ± 9.32	9.41 ± 12.31	7.27 ± 1.54
Set E	45.84 ± 352.61	73.73 ± 649.34	11.21 ± 23.89	34.56 ± 268.48	7.76 ± 1.37
(b) Power spectrum error					
Set A	2.53 ± 1.60	2.64 ± 1.76	2.20 ± 1.37	2.13 ± 1.65	0.44 ± 0.28
Set B	3.88 ± 4.57	4.30 ± 6.19	3.38 ± 4.89	3.34 ± 4.87	0.74 ± 0.92
Set C	4.68 ± 3.91	4.83 ± 4.19	4.30 ± 3.42	3.30 ± 2.44	0.66 ± 1.02
Set D	6.03 ± 12.84	6.14 ± 12.56	5.86 ± 11.01	5.09 ± 15.31	0.59 ± 0.54
Set E	4.64 ± 5.48	5.76 ± 13.29	5.02 ± 17.35	4.58 ± 9.67	0.87 ± 0.37
(c) Hurst component error					
Set A	0.09 ± 0.06	0.19 ± 0.11	0.08 ± 0.05	0.17 ± 0.11	0.003 ± 0.02
Set B	0.08 ± 0.07	0.07 ± 0.06	0.08 ± 0.05	0.20 ± 0.13	0.004 ± 0.0012
Set C	0.11 ± 0.07	0.13 ± 0.07	0.08 ± 0.05	0.16 ± 0.09	0.028 ± 0.019
Set D	0.06 ± 0.06	0.09 ± 0.07	0.07 ± 0.06	0.14 ± 0.10	0.009 ± 0.0027
Set E	0.18 ± 0.10	0.15 ± 0.11	0.10 ± 0.07	0.17 ± 0.11	0.023 ± 0.011

(a) Of MAE error (where the result of Random HR, Small world HR, Random Kuramoto, Small world Kuramoto are taken from Nguyen et al. (2020). And Hopf-hidden network is our proposed model (rightmost in the table)). (b) Of Power spectrum error MAE error [where the result of Random HR, Small world HR, Random Kuramoto, Small world Kuramoto are taken from Nguyen et al. (2020). And Hopf-hidden network is our proposed model (rightmost in the table)]. (c): Hurst component error MAE error [where the result of Random HR, Small world HR, Random Kuramoto, Small world Kuramoto are taken from Nguyen et al. (2020). And Hopf-hidden network is our proposed model (rightmost in the table)].

### 3.4 Sensitivity analysis

In our Model, there are several parameters that can be varied and the effect on the network performance can be assessed. For this purpose, we consider four parameters:

Oscillator amplitude (*μ*).
Coupling coefficient ( $\;\xi_{w}$ ), in Equation 1f.
Beta (*β*) parameter in the Hopf oscillator.
Additional Hidden Layer.

**Figure 9:**
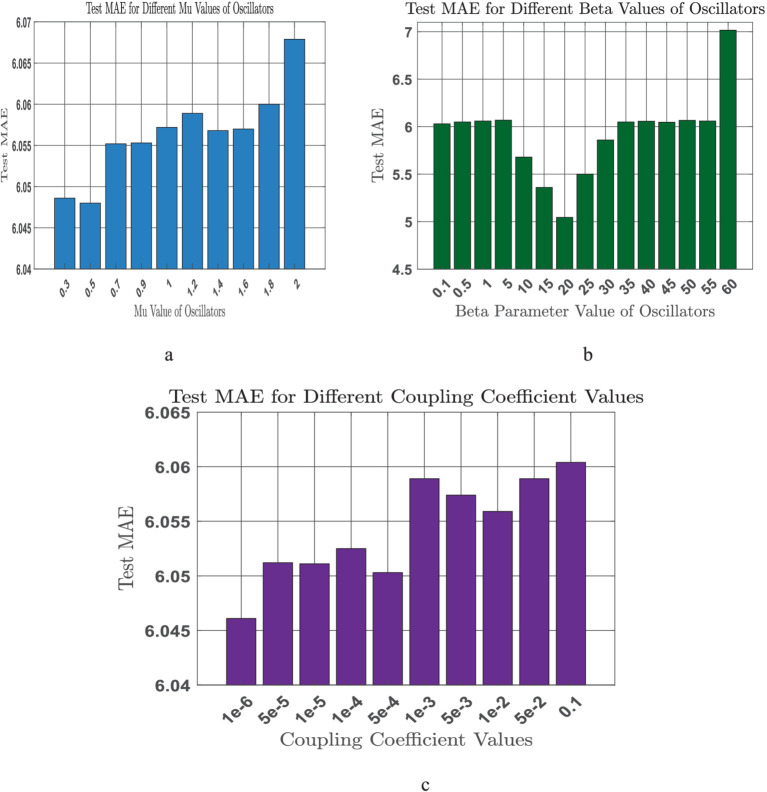
**(a-c)** Sensitivity analysis on tunable model parameters [**(a)** Oscillator amplitude (*μ*); **(b)** Coupling coefficient $\;(\xi_{w});$ **(c)** Beta **(𝜷)**].

The oscillator amplitude parameter (μ) is varied over the range of (0.5 to 2), and the corresponding mean absolute error is shown below (Figure 9a). We observe that with an increase of mu(μ) over the given range no such significant change is visible in the output error (see Figure 9b). This probably because any change in μ is offset by an compensating change in the weights from the oscillators to the hidden layer, so as to produce the same output error.

Also, we next vary the coupling coefficient magnitude $\;(\xi_{w})$ , which scales the coupling among the oscillators., We have seen with the increase of coupling coefficient MAE error also increased (Figure 9a). This probably because any change in the coupling coefficient is offset by an compensating change in the coupling weights among the oscillators, so as to produce the same output error.

We change the (β) parameter over the range of (0.1 to 60), and the corresponding mean absolute error is shown below (Figure 9c). We observe that the model gives optimal error in the neighborhood of beta = 20. Also, at the end we have optimized the model parameters.

Also, we have done a comparison analysis with the addition of another more hidden layer (Table 6), and we observed that there is no significant change in model training and testing performances.

**Table 6:** Network performances after adding an additional hidden layer.

Network parameters	Training MAE	Testing MAE
*Two Hidden Layer:* Number of oscillators: 250, Learning rate: 0.001 1st Hidden layer: 50-tanh activation function 2nd hidden layer: 20-tanh activation function Epochs: 10000	0.03	6.93–8.26
*Single Hidden layer*: by network architecture mentioned in Table 3	0.01–0.02	5.56–6.67

### 3.5 Spherical shell model

In this study we create a spherical (radius 85 mm obtained from EEGLab) on which the electrodes are placed. Underneath this spherical shell, we place two more spherical surfaces forming a hollow spherical shell, of inner radius (r_1 = 70 mm) and outer radius (r_2 = 75 mm), within which the oscillators are distributed. Within this spherical shell we distribute 1,000 Hopf oscillators. Sample locations for a few channels in spherical (Table 7) are shown. (Also, sample locations of electrodes in Cartesian coordinate has been shown in Supplementary Table S4).

**Table 7:** Sample location in spherical coordinate system from EEGLAB.

Channel name	Spherical Th (*θ*)	Spherical Phi(Ф)	Radius(r) (mm)
F1	0	44.392	85
Fz	23.493	39.775	85
F2	−23.493	39.819	85

**Figure 10:**
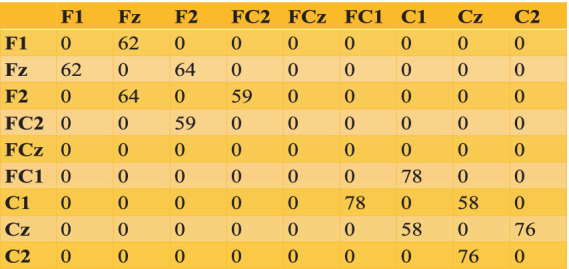
Pair of electrodes and their shared oscillators.

Using a similar distance threshold ( $\xi_{1}=32$ ) defined in Equation 6a, we allocate oscillators to various electrodes (Supplementary Table S5). The number of shared oscillators between pairwise electrodes are given in Figure 10. Connectivity among oscillators is determined by the threshold value $\left(\xi_{2}=10\right)$ .

**Figure 11:**
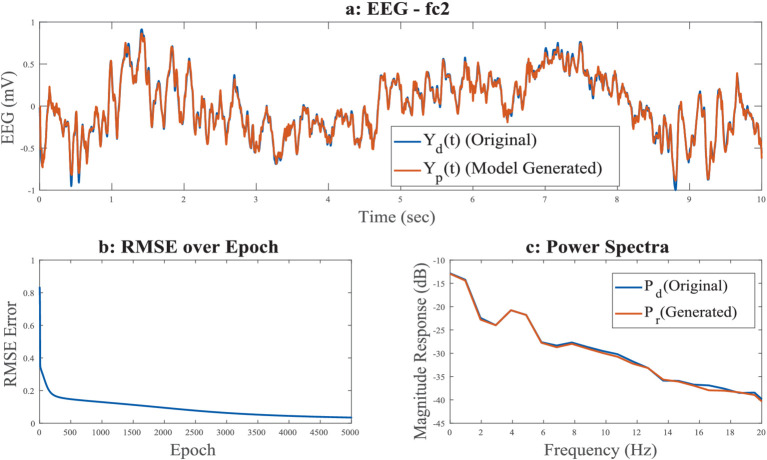
**(a–c)** Time series of reconstructed signal and the desired signal for FC2 channel among 8 channels in a spherical shell, during training stage; **(b)** RMSE error w.r.t. training epochs; **(c)** power spectrum of desired and reconstructed signal, $Y_{d}(t)=$ desired EEG, $Y_{p}(t)=$ predicted EEG, $P_{d}=$ desired EEG power, $P_{r}=$ desired EEG power.

We can see the oscillator distribution is unequal for the rectangular grid and the spherical shell. That is because of their geometrical shape. In the case of the rectangular grid, a total of 972 oscillators are distributed over three layers; each unit of the rectangular grid consists of one oscillator, whereas in the spherical shell, 1,000 oscillators are randomly distributed over a spherical surface (see Figure 11).

In the case of the rectangular grid, no proper electrode geometry was followed, all 8 electrodes are in same plane, and oscillators distributed over channels are not far from each other (see Supplementary section 10). But in the case of the spherical shell, the electrode layer also has a spherical geometry with the same curvature as that of the oscillator layer, and oscillators distribution within the layer is random. For example, channel Cz is estimated by 219 number of oscillators and its nearest channel C2 is estimated by 149 oscillators. One reason might be the same fixed threshold is used to calculate the number of oscillators belonging to each channel. Also, in comparison with a rectangular grid, a larger number of oscillators are assigned here.

**Figure 12:**
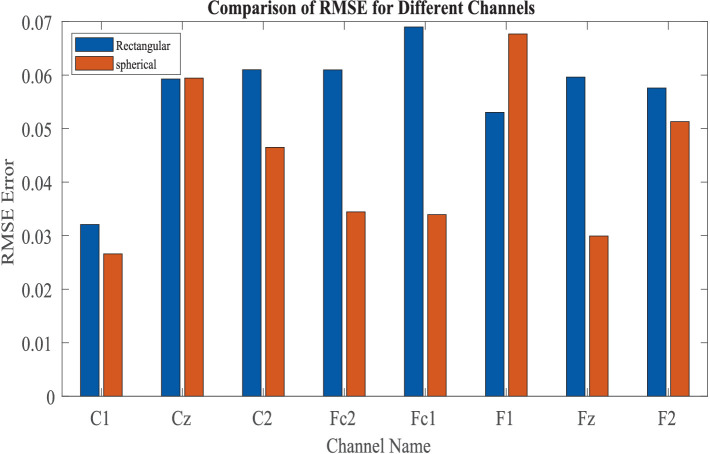
Comparison of reconstruction error in between “Spherical shell” and “rectangular grid” model.

We have evaluated the reconstruction errors bar plot across eight channels using two model architectures: (a) Rectangular and (b) Spherical (Figure 12). The results indicate that the Spherical model provides a better overall fit. However, for the F1 channel, the rectangular model achieves higher reconstruction accuracy.

## 4 Discussion

In the present study, we model a 56-channel EEG signal with a network of oscillatory neurons. The proposed network is able to model (both reconstruction and prediction) whole-brain EEG data. The network is able to successfully predict test signals over a significant duration beyond the training duration (5 s), and is able to retain properties (Hurst component and Higuchi fractal dimension) of the actual EEG signals over the frequency band of interest.

In the present study, insertion of a hidden layer between the oscillator layer and the output layer is proven to improve reconstruction quality (Supplementary section 7) significantly. This is because when there was no hidden layer, the output signal was essentially approximated by a finite set of sinusoidal signals represented by the oscillators of the input layer. But once we applied the hidden layer in our 2nd network, those sinusoids pass through the nonlinear sigmoid functions of the hidden layer. Even when a single sinusoid passes through a nonlinear sigmoid, we can get all the infinite harmonics. Furthermore, when a mixture of sinusoids is passed through a sigmoid function, we get the harmonics not only of the original frequencies but the harmonics of all the mixtures (e.g., $\pm n_{i}\omega_{i}\pm n_{j}\omega_{j}\pm n_{k}\omega_{k}$ ). Hence, the hidden layer is expanding the spectrum that is available at the output layer. Thus, the finite number of frequencies available when we did not apply a hidden layer suddenly explode to an infinite set of frequencies available when we apply a hidden layer.

In comparison with Nguyen et al. (2020), where 3,000 neurons used to simulate EEG data whereas in our model the network with only 250 Hopf oscillators was able to predict more accurately even without using any data reduction techniques like the PCA.

Our proposed method can predict EEG Data in the order of 5 s (2,500 Data points). The predicted signal has good agreement with actual EEG with respect to power spectrum, Hurst exponent (Supplementary material) and complexity measure (Higuchi fractal dimension) (Supplementary material) which are the key results of our study. A potential application of our network can be synthetic EEG generation for research and education.

Also, we have successfully demonstrated spatial localization architecture for oscillator reservoirs. The performance of spatially arranged oscillators with a hidden layer is good, as demonstrated by RMSE values. However, in our spatial geometry of oscillators, we only considered locally connected regions. Also, real structural data can be implemented on this proposed model to realize a large-scale TVB type of model (Sanz Leon et al., 2013; Al-Hossenat et al., 2019). In the future, we can easily expand our model to a real MRI-based surface where oscillators are placed according to structural-functional connectivity nodes. Thus, the problem regarding the unequal distribution of oscillators in the case of “spherical shell” geometry can be eliminated.

Each EEG channel was modeled by a single Hindmarsh Rose neuron in an ADHD study (Ansarinasab et al., 2023); since EEG represents the collective activity populations of neurons, in the present model, each EEG channel is modeled by a network of oscillators. Also, a graph-based brain topology was used in the same study (Ansarinasab et al., 2023), whereas in the proposed model, we distribute the oscillators spatially as per two different geometries: rectangular and spherical. It is very difficult to predict non-stationary signal like EEG beyond the training duration, however, deep learning generative model [GAN type model (Panwar et al., 2020; Aznan et al., 2019a)] requires a huge training sample, that problem also can be solved by our proposed oscillatory generative model.

While both works utilize, like a good number of existing models of brain dynamics (Ghorbanian et al., 2014; Ghorbanian et al., 2015a; Ghorbanian et al., 2015b; Szuflitowska and Orlowski, 2021; Babloyantz et al., 1985; Rankine et al., 2006; Burke and de Paor, 2004; Ren et al., 2017; Nguyen et al., 2020; Logothetis et al., 2001; Cabral et al., 2023; Breakspear, 2017; Deco et al., 2015; Deco et al., 2017; Deco et al., 2021; Luppi et al., 2022; López-González et al., 2021; Deco and Kringelbach, 2014; Ponce-Alvarez and Deco, 2024), our case, network of Hopf oscillators as the basic source of oscillations, the specific methods and the outcomes of the two studies are significantly different.

Our group recently developed a similar network using fMRI signals. In that work, Bandyopadhyay et al. (2023) demonstrated a strong alignment between predicted and empirical functional connectivity (FC), as validated through graph-theoretical analysis. Authors have shown that structural damage resulted in cascading disruptions in static and dynamic FC patterns. Computational interventions revealed that optimizing the coupling coefficient (*μ*) could restore functional integrity. This in silico perturbation study further highlighted how targeted parameter adjustments could compensate for structural degradation, providing insights into potential therapeutic applications. These findings underscore the model’s potential in understanding and addressing disruptions in brain network dynamics.

However, a key distinction between our proposed study and that of Bandyopadhyay et al. (2023) lies in the testing regime. While their work primarily focused on training, signal reconstruction, and the connection between structural and functional connectivity, they did not discuss the network’s behavior beyond training. In contrast, our proposed network can retain learned patterns beyond the training signal. Additionally, we show that the network-generated signals preserve key properties in both the time and frequency domains. Furthermore, we introduced a spherical shell model based on the 10–20 EEG electrode system, enabling EEG signal reconstruction while utilizing shared oscillators’ natural frequencies and phases. We also explored optimal network parameters to achieve the best fit, enhancing the model’s reliability and performance. Data augmentation for EEG has gained significant research attention in recent years (Torma and Szegletes, 2025; Aznan et al., 2019b; Kalaganis et al., 2020; Hartmann et al., 2018). Collecting EEG data has several challenges, primarily due to the strict requirements of the environment and the variability in subjects’ psychological and physiological conditions. Due to the limited accessibility of the highly dense EEG data, applying several deep learning models is challenging. To address this issue, data augmentation techniques have emerged as a viable solution. This deep oscillatory neural generative model offers strong potential for synthesizing realistic EEG data. Additionally, we have included a comparison table (Table 8) comparing our method with that of Bandyopadhyay et al. (2023).

**Table 8:** Comparison with Bandyopadhyay et al. (2023).

	Bandyopadhyay et al. (2023)	Proposed work
Type of model	Hopf oscillator with hidden layer and two-stage training	Hopf oscillator with hidden layer and two-stage training
Signal used in the model	BOLD-fMRI with 160 ROI, each ROI has been represented by one oscillator	Sleep EEG of different sleep stages-62 channels, Each EEG electrode has been assigned oscillators based on the threshold.
Lateral connection/Method	Structural connectivity was used.	No such structural connectivity was used.
Spatial arrangement of oscillator	Oscillators are placed based on structural connectivity. However, the connection strength among the oscillators was taken from real brain structural connectivity.	Spatial arrangements of oscillators were provided based on real brain head surface (10–20 EEG electrode placement rule). Individual electrodes have a bunch of oscillators, and nearby two electrodes have a few common oscillators. Their learned frequency and phase have also been used.
Analysis matrix	The correlation coefficient between empirical and simulated signals has been calculated	MAE error Power spectrum error Hurst component error between empirical and simulated signal has been calculated
Key findings	The impact of structural information loss on functional information due to disease conditions was evaluated using the correlation coefficient on simulated and empirical functional connectivities (FCs).	This approach can be helpful for synthetic EEG generation. Future predictions of the EEG signal have been discussed beyond training. The predicted signal strongly agrees with the actual EEG regarding power spectrum, Hurst exponent, and sensitivity analysis. Statistical tests reveal that the predicted signal closely matches the actual EEG signal. Additionally, we have demonstrated the specific tuning parameter values that influence these results (μ, ζ w, 𝜷).
Model validation	Paris dataset	Our proposed model was compared with the publicly available BONN Dataset.

Baseline models Ren et al. (2017), Ghorbanian et al. (2015a), and Ghorbanian et al. (2015b) utilized the same modeling and training methodologies. Our assessment of existing EEG modelling approaches highlights significant differences and enhancements in our proposed framework (Table 9). This structured comparison emphasizes our framework’s advancements in achieving a more biologically plausible network with improved computational traceability.

**Table 9:** Summary report between different models with the current model.

Works	Model used	Trainable parameters	Output	Remarks
23	HR neuron, small scale network	Trainable parameters: Coefficient of oscillator’s activation; Using least square fitting rule, linear and nonlinear coupling, coupling coefficients are not trained	Training error:(0.04–06) Testing: No, (4 EEG channel)	Time series fitting is not good. The power spectrum does not match. There is no training for coupling coefficients.
24	HR and Kuramoto neuron, random and small world network	Trainable parameters: Coefficient of oscillator’s activation; Using least square fitting rule, linear and nonlinear coupling, coupling coefficients are not trained	Training error:(0.02–05) Testing: Yes (7.95 ± 6.69-MAE error), 4 EEG channel	Hurst component: In a few cases, it matches well Power spectrum: In a few cases, it matches well With more computational complexity (A high number of neurons (400)and PCA being applied over there), testing performances are unsatisfactory.
16	Duffing van dar poll oscillator	Global optimization search method (*μ,* $K_{i}$ are trained) power spectrum and Shannon entropy used for cost function	Time series prediction: not mentioned Power spectrum: 0.502 ± 0.091 (model); 0.490 ± 0.100 (Empirical):	EC Alpha band Delta, beta, and gamma band power is not matching(EC) Alpha, beta, and gamma power do not match (EO), and Shanon entropy does not match (EO).
18	10 coupled Harmonic oscillator model and 2 coupled Duffing oscillator model	Global optimization search method (*μ,* $K_{i}$ are trained), power spectrum used for cost function, linear coupling	Shannon entropy 1.80 ± 0.08(EC EEG) and 1.92 ± 0.08(model output), 1.71 ± 0.11(EO EEG) and 1.57 ± 0.15 (model output)	EC-theta, alpha EO-alpha, beta band power matches well Noise driven model may cause the less predictable property
68	Hopf oscillatory Network	Autoregressive method, Omega values are not trained, 4 Hopf oscillators are fixed normalized frequencies (1,3.194,5.833, 12.22),simulations are done on R-K methods	Modeling of single channel EEG data (Oz)	amplitude statistics appear to be in good agreement with the actual EEG The 2D phase plots of the model and actual EEG are also quite similar
Our proposed work	Hopf oscillatory model	Nobel power coupling is introduced, Hebb’s rule trains the angle of power coupling, feed word weights are also trainable, and natural frequencies are trainable.	Whole brain 56 EEG electrodes are model for all sleep stages Evaluation method: MAE error Power spectrum error Hurst component error Between empirical and simulated signals has been calculated	The best error we achieved during testing which surplus previously reported literature (Nguyen et al., 2020): MAE: 5.71 ± 1.92 Power spectrum error: 0.44 ± 0.28 Spatial localization of oscillators. Combinations of oscillatory and sigmoid neurons are explored.

Additionally, we introduced a spherical shell model based on the 10–20 electrode geometry, enabling the reconstruction of EEG signals. In this model, the natural frequencies and phases of the shared oscillators were utilized effectively. Furthermore, we explored and identified optimal network parameters to achieve the best fit, ensuring superior signal reconstruction and validation performance.

Future efforts will be directed to developing the current model into a more realistic model of sleep dynamics. In the current modelling approach, separate networks are trained to produce EEG signals of various sleep stages and the waking stage. However, in a more authentic model of sleep, it is desirable to generate the various stages in a single model and explicitly demonstrate the transitions from one stage to the next. Ideally, such a model will show the sleep–wake cycle at a longer or diurnal time scale, and also the entire architecture of sleep substages within the 8-h long sleep stage. The model also will permit a minimal representation of the main neural substrates of sleep regulation such as the Suprachiasmatic Nucleus (SCN), hypothalamic and thalamic nuclei and the neuromodulatory systems involved in sleep regulation, and the Reticular Activating System (RAS). Our approach combines empirical EEG data with a mathematical model to create virtual representations of the brain’s oscillatory dynamics. It can help to explore the impact of neuronal excitability, synaptic plasticity, and network connectivity on sleep stage transitions (Tatti and Cacciola, 2023).

In the future sleep model that we envisage, the interactions among the various subcortical circuits and neural systems produce the subrhythms of sleep, which, acting on the cortex, generate the EEG activity patterns characteristic of the relevant sleep stage. In this paper, we primarily focus on sleep EEG. However, our model is a general-purpose, universal framework that can be applied to any EEG dataset. To validate its universality, we tested the model on the publicly available BONN dataset, demonstrating improved performance compared to the previously reported study (Nguyen et al., 2020).

## Data Availability

The raw data supporting the conclusions of this article will be made available by the authors, without undue reservation.
