# Supplementary material for: Modeling of whole brain sleep electroencephalogram using deep oscillatory neural network
Source: Front Neuroinform. 2025 May 14;19:1513374. doi: 10.3389/fninf.2025.1513374 (PMC12116487; doi:10.3389/fninf.2025.1513374)
Supplement: Supplementary file 1 [file Data_Sheet_1.pdf]

Supplementary Material

Supplementary material

Sections:

1. The polar coordinate representation

2. Git hub link (source code)

3. Derivation the Hidden layer

4. Result: with 1st phase of training

5. Result: with 2nd phase of training

6. Result: after inserting a hidden layer

7. Effect of hidden layer size

8. Evaluation of phase locking value

9. Generation of EEG data

10. Spatial distribution of oscillator

11. Evaluation of Hurst component

12. Statistical test of HFD

13. Statistical test on PSD

14. Spherical shell model

15. Description on BONN Dataset

16. Comparison Table

---

1. The polar coordinate representation of eqn. 1(c) is:

$$\dot{r}_i = \left(\mu + \beta {r_i}^2\right) r_i + \sum_{j=1 \;\ni\; j\neq i}^{N} A_{ij} r_j^{\frac{\omega_i}{\omega_j}} \cos \omega_i \left(\frac{\theta_j}{\omega_j} - \frac{\theta_i}{\omega_i} + \frac{\emptyset_{ij}}{\omega_i \omega_j}\right) \tag{1}$$

$$\dot{\theta}_i = \omega_i + \sum_{j=1 \;\ni\; j\neq i}^{N} A_{ij} \frac{r_j^{\frac{\omega_i}{\omega_j}}}{r_i} \sin \omega_i \left(\frac{\theta_j}{\omega_j} - \frac{\theta_i}{\omega_i} + \frac{\emptyset_{ij}}{\omega_i \omega_j}\right) \tag{2}$$

where r and θ are the state variables, $(\sqrt{\frac{\mu}{\beta}})$ is the amplitude of oscillation and β is a bifurcation parameter. $\mu = 1$, $\beta = -20$. A_{ij}, is the magnitude of complex coupling coefficient, $(A_{ij} \ll 1)$, θ_{ij} is angle of complex coupling coefficient, $\emptyset_i$ and ω_i and are the ith oscillator's phase and intrinsic frequency respectively.

2. Github link (Source Code)

I have added the source code of our model, which will help during reproducibility or future work.

Link: https://github.com/sayanGh-lab/Oscillatory-Generative-model

3. Hidden layer equation

(learning rates: η_h =0.001; η_o =0.001).

$$\dot{r_i} = (\mu + \beta r_i^2) r_i + \sum_{j=1 \ni j \neq i}^{N} A_{ij} r_j^{\frac{\omega_i}{\omega_j}} cos\ \omega_i \left(\frac{\theta_j}{\omega_j} - \frac{\theta_i}{\omega_i} + \frac{\emptyset_{ij}}{\omega_i \omega_j} \right) \quad (5)$$

$$\dot{\theta}_i = \omega_i + \sum_{j=1 \ni j \neq i}^{N} A_{ij} \frac{r_j^{\frac{\omega_i}{\omega_j}}}{r_i} sin\ \omega_i \left(\frac{\theta_j}{\omega_j} - \frac{\theta_i}{\omega_i} + \frac{\emptyset_{ij}}{\omega_i \omega_j} \right) \quad (6)$$

Convert polar coordinate equation (r and θ) to complex domain according to ($z = re^{i\theta}$)

$$\mathrm{Z_r} = \mathrm{real}(\mathrm{rarr2} * \mathrm{e}^{\mathrm{i*tharr2}}) \quad (7)$$

$$\mathrm{Z_i} = \mathrm{imag}(\mathrm{rarr2} * \mathrm{e}^{\mathrm{i*tharr2}}) \quad (8)$$

$$\mathrm{Z} = \mathrm{Z_r} + \mathrm{iZ_i} \quad (8a)$$

Where Z is the complex domain activation of hopf oscillator. $\mathrm{Z_r}$ is the real part of oscillator activation, $\mathrm{Z_i}$ is the imaginary part of oscillator activation.

Forward propagation:

$$\mathrm{W_{lk}^{f1}} = \mathrm{W_{lk,R}^{f1}} + \mathrm{iW_{lk,I}^{f1}} \quad (9)$$

Where $\mathrm{W_{lk}^{f1}}$ is the feedforward weight between oscillatory layer to 1st hidden layer. These feedforward weights are initialized in complex domain. It has both real ($\mathrm{W_{lk,R}^{f1}}$) and imaginary part ($\mathrm{W_{lk,I}^{f1}}$).

$$\mathrm{W_{ml}^{f2}} = \mathrm{W_{ml,R}^{f2}} + \mathrm{iW_{ml,I}^{f2}} \quad (10)$$

Where $\mathrm{W_{ml}^{f2}}$ is the feedforward weight between 1st hidden layer to output layer. These feedforward weights are initialized in complex domain. It has both real ($\mathrm{W_{ml,R}^{f2}}$) and imaginary part ($\mathrm{W_{ml,I}^{f2}}$).

$$\mathrm{n_l^{Hf}} = \sum_k \mathrm{W_{lk}^{f1} Z_k} = \sum_k \left(\mathrm{W_{lk,R}^{f1} Z_{k,R}} - \mathrm{W_{lk,I}^{f1} Z_{k,I}} \right) + \mathrm{i} \sum_k (\mathrm{W_{lk,I}^{f1} Z_{k,R}} + \mathrm{W_{lk,R}^{f1} Z_{k,I}}) \quad (11)$$

Where $\mathrm{n_l^{Hf}}$ is the product of complex oscillatory activation ($\mathrm{Z_k}$) and $\mathrm{W_{lk}^{f1}}$.(eqn. 12) It has both real($\mathrm{n_{l,R}^{Hf}} = \sum_k \left(\mathrm{W_{lk,R}^{f1} Z_{k,R}} - \mathrm{W_{lk,I}^{f1} Z_{k,I}} \right)$) and imaginary part ($\mathrm{n_{l,I}^{Hf}} = \sum_k (\mathrm{W_{lk,I}^{f1} Z_{k,R}} + \mathrm{W_{lk,R}^{f1} Z_{k,I}})$).

$$\mathrm{n_l^{Hf}} = \mathrm{n_{l,R}^{Hf}} + \mathrm{in_{l,I}^{Hf}} \quad (12)$$

Both $n^{Hf}_{l,R}$ and $n^{Hf}_{l,I}$ are passed through tanh function separatedly. Real part of sigmoid activation ($X^{Hf}_{l,R} = f^h_R(n^{Hf}_{l,R})$: *real part of* 1*st* tanh *activation*) and ($X^{Hf}_{l,I} = f^h_I(n^{Hf}_{l,I})$) (: imaginary part of 1st tanh activation) and X^{Hf}_l is the total activation of 1st hidden layer.(eqn. 13 & eqn, 14).

$$X^{Hf}_l = f^h_R(n^{Hf}_{l,R}) + if^h_I(n^{Hf}_{l,I}) \quad (13)$$

$$X^{Hf}_l = X^{Hf}_{l,R} + iX^{Hf}_{l,I} \quad (14)$$

Where n^o_m is the product of 1st hidden layer activation (X^{Hf}_l) and W^{f2}_{ml}.(where W^{f2}_{ml} is the 1st hidden to output node weight, $W^{f2}_{ml,R}$, $W^{f2}_{ml,I}$ are the real and imaginary part of W^{f2}_{ml})(eqn. 15) It has both real($n^o_{m,R} = \sum_l (W^{f2}_{ml,R}X^{Hf}_{l,R} - W^{f2}_{ml,I}X^{Hf}_{l,I})$) and imaginary part ($n^o_{m,I} = \sum_l (W^{f2}_{ml,I}X^{Hf}_{l,R} + W^{f2}_{ml,R}X^{Hf}_{l,I})$).

$$n^o_m = \sum_l W^{f2}_{ml}X^{Hf}_l = \sum_l (W^{f2}_{ml,R}X^{Hf}_{l,R} - W^{f2}_{ml,I}X^{Hf}_{l,I}) + i\sum_l (W^{f2}_{ml,I}X^{Hf}_{l,R} + W^{f2}_{ml,R}X^{Hf}_{l,I}) \quad (15)$$

$$n^o_m = n^o_{m,R} + in^o_{m,I} \quad (16)$$

At output node (eqn. 17) we consider the only real part of n^o_m, which is $n^o_{m,R}$. And also at output layer we have tanh neuron.

$$Y_m = f^o_R(n^o_{m,R}) \quad (17)$$

Backpropagation: The complex domain backpropagation has been adapted from [1].

Loss at every time step,

$$L(t) = \frac{1}{2}(O_m(t) - Y_m(t))^2 \quad (18)$$

Where , $O_m(t)$ is the desired (target) signal.

$$\frac{\partial L}{\partial W^{f2}_{ml,R}} = (O_{m,R} - Y_{m,R})f^{o\prime}_R X^{Hf}_{l,R} \quad (19)$$

$$\frac{\partial L(t)}{\partial W^{f2}_{ml,I}} = (O_{m,R} - Y_{m,R})f^{o\prime}_R X^{Hf}_{m,I} \quad (20)$$

$$\frac{\partial L(t)}{\partial W^{f1}_{lk,R}} = (-1)\sum_m (O_{m,R} - Y_{m,R})f^{o\prime}_R (W^{f2}_{ml,R}f^{h\prime}_R Z_{k,R} - W^{f2}_{ml,I}f^{h\prime}_I Z_{k,I}) \quad (21)$$

$$\frac{\partial L(t)}{\partial W^{f1}_{lk,I}} = \sum_m (O_{m,R} - Y_{m,R})f^{o\prime}_R (W^{f2}_{ml,R}f^{h\prime}_R Z_{k,I} + W^{f2}_{ml,I}f^{h\prime}_I Z_{k,R}) \quad (22)$$

Rewrite the Activation function:

Sigmoidal activation function:

$$\boldsymbol{f}(\mathrm{x}, \mathrm{a_k}, \mathrm{c_k}) = \frac{1}{1+\exp^{(-\mathrm{a_k}(\mathrm{x}-\mathrm{b_k}))}} \quad (23)$$

Put , $\mathrm{a_k} = 0.5, \mathrm{b_k} = 0,$

$$2\ \mathrm{f}(\mathrm{x}, \mathrm{a_k}, \mathrm{c_k}) - 1 = \frac{1-\exp^{-0.5\mathrm{x}}}{1+\exp^{0.5\mathrm{x}}} = \tanh\ (\mathrm{x}/2) \quad (24)$$

4. Result of 1st phase of Training

Time series reconstruction with real alpha value(0.2) and corresponding power spectrum density is shown in Fig. 1(a). And most importantly how coupled hopf oscillatory network adapt signal frequencies is shown in 1(b).

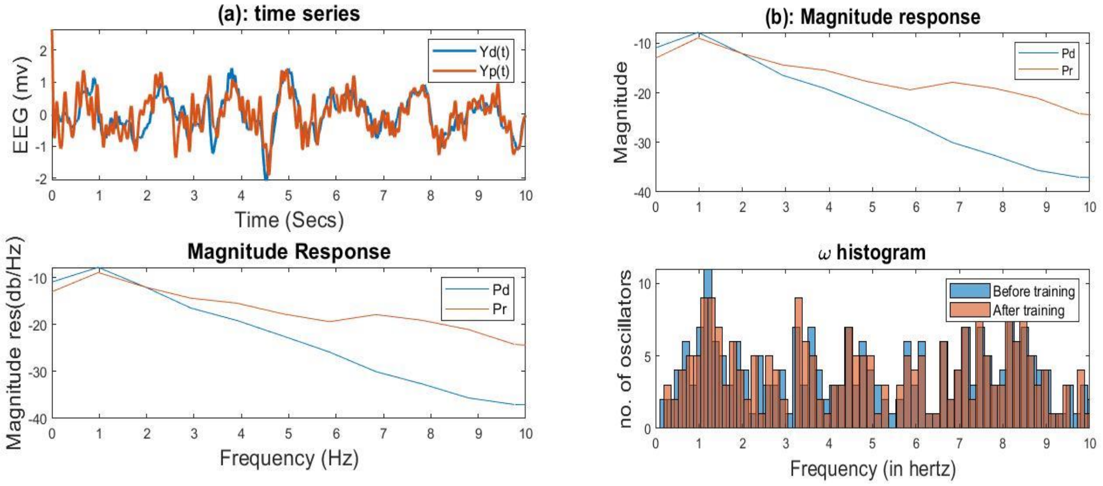

Supplementary Figure 1: (a) Time series reconstruction (training), (b) learning the intrinsic frequencies. Y_d(t)=desired EEG, Y_p (t)= reconstructed EEG, Pr=power spectrum of reconstructed signal, Pd=power spectrum of desired signal

5. Result of 2nd stage of training

Time series reconstruction with complex alpha value and corresponding power spectrum density is shown in Fig. 2(a-d). Where trained oscillator frequencies are taken from 1st stage of training.

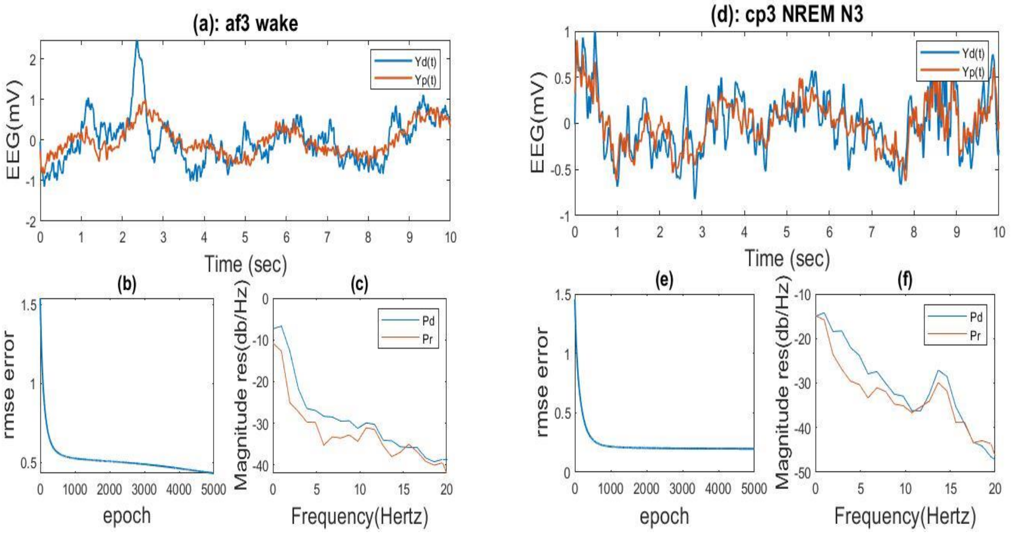

Supplementary Figure 2: (a),(d)- Time series of reconstructed and desired signal for af3 (wake) and cp3 (NREM N3) channels, (b),(c)-RMSE variation over training epochs, (c),(f)-power spectrum of desired and reconstructed signal, $Y_d(t)$ = desired EEG, $Y_p(t)$ = reconstructed EEG, P_d = desired EEG power spectra, P_r = reconstructed EEG power spectra.

6. Result after inserting hidden layer

Training result after applying hidden layer:

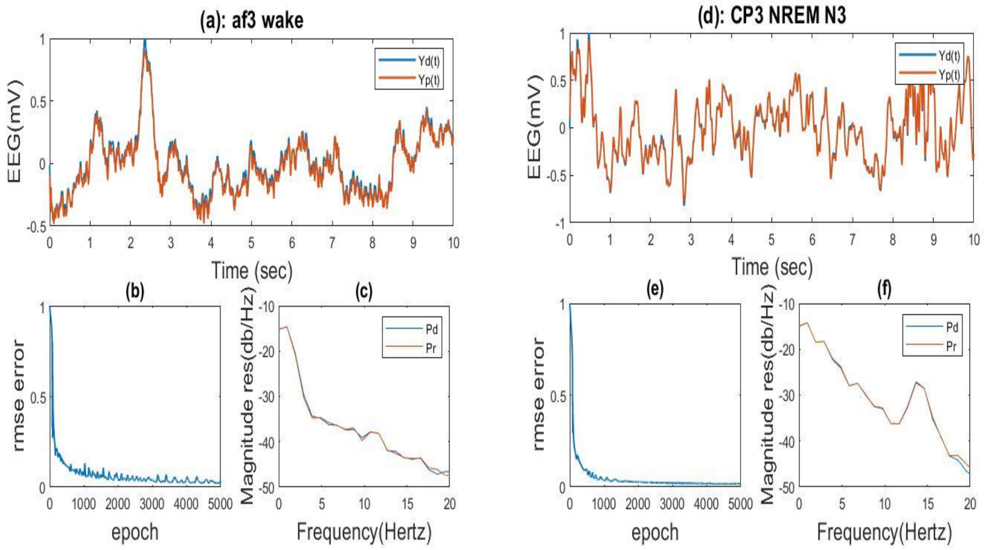

Supplementary Figure 3: (a) (–) - Time series of reconstructed and desired signals for af3 (wake) and cp3 (NREM N3) channel during training, (©(e)-RMSE over training, (c), (f)-power spectrum of desired and reconstructed signal, $Y_d(t)$ = desired EEG, $Y_p(t)$ = reconstructed EEG, P_d = desired EEG power spectra. P_r=reconstructed EEG power spectra.

Comparison between reconstruction accuracy (during training) between without hidden layer and with hidden layer.

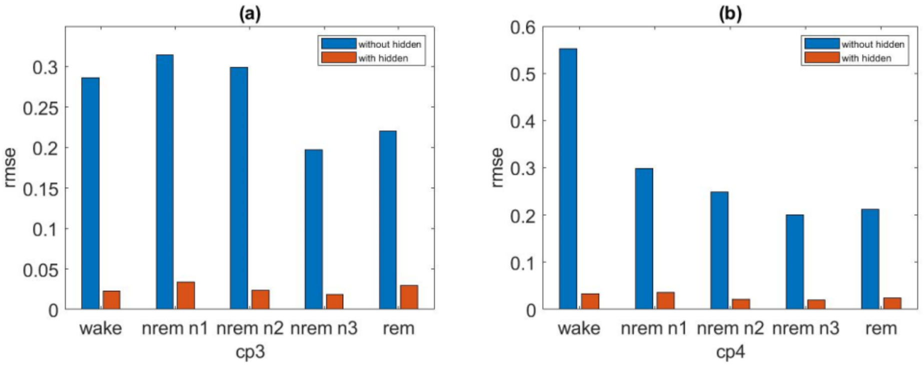

Supplementary Figure 4: (a)- ‘CP3’,(b)- ’CP4’ bar plot of RMSE error(Training RMSE) comparing with ‘without hidden layer’ & ‘with hidden layer’ of four different electrodes for all five sleep stages.

7. Effect of the hidden layer size

In the previous section, we observed enhancement of model performance after adding additional a hidden layer which consists 100 sigmoidal neurons. Here we vary the number of hidden neurons in the hidden layer and compare the corresponding reconstruction RMSE (fig. 5) during training. As expected, increasing the hidden layer size reduced RMSE over the range of hidden layer sizes shown in fig. 13. However, further increase might cause overfitting and an increase in RMSE.

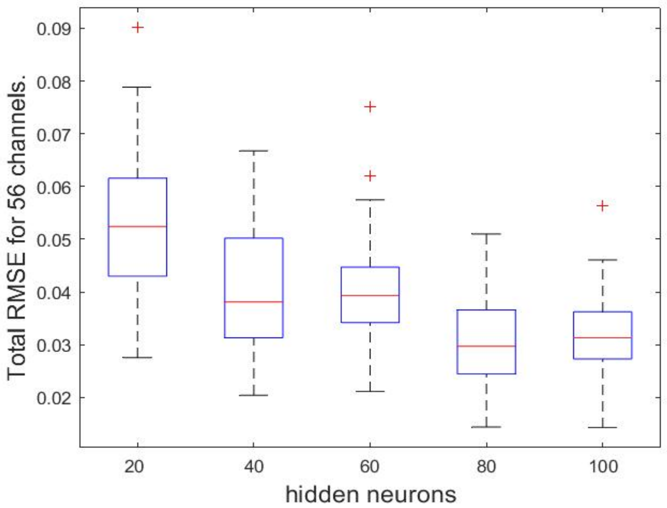

Supplementary Figure 5: Comparing the effect of hidden layer size to reconstruct 56 EEG electrodes (during training)

8. Phase locking value (PLV)

The Functional matrix(FC) represents as a influential tool for understanding brain dynamics and brain networks [5-6]. Analysing the FC of model predicted signal and empirical signal is a promising benchmark to evaluate model performances [7-8].

PLV measures the instantaneous phase difference, calculated using the Hilbert transform, between two narrow band signals coming from two channels. The range of the PLV value is between [0, 1], where 0 means no phase synchrony and 1 means perfectly synchronized [2].

PLV is calculated using

$$PLV(i,k) = \left|\frac{1}{N}\sum_{t=1}^{N} e^{i(\Delta\emptyset)}\right| \tag{25}$$

For N time points, it calculates the average of N unit phasors that represent phase differences**.**

$$\Delta\emptyset = \emptyset_1 - \emptyset_2 \tag{26}$$

After calculating the phase, computation of PLV is done by using eqn. 4(a). Here we estimate Phase Locking Value (PLV) of experimental EEG data as well as model predicted EEG data.

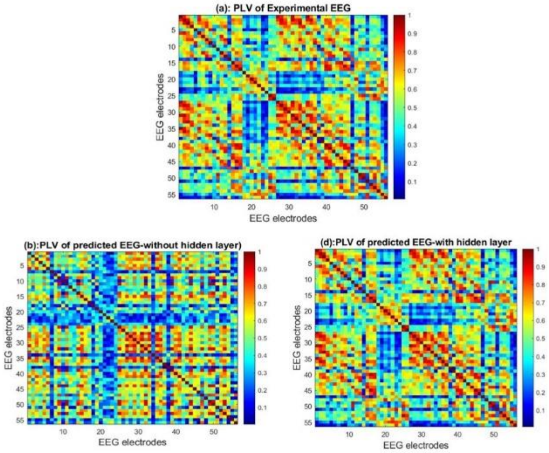

Supplementary Fig 6: (a): FCM for experimental EEG Data, (b): FCM of network predicted signal using ‘without hidden layer network ‘, (d) FCM of network predicted signal using ‘with hidden layer network’.

9. Generation of EEG Data

$$\dot{r_i} = \left(\mu + \beta {r_i}^2\right) r_i + \sum_{j=1}^{N} {}_{\ni\, j\neq i} A_{ij} r_j^{\frac{\omega_i}{\omega_j}} cos\ \omega_i \left(\frac{\theta_j}{\omega_j} - \frac{\theta_i}{\omega_i} + \frac{\emptyset_{ij}}{\omega_i\omega_j}\right) \quad (27)$$

$$\dot{\theta}_i = \omega_i + \sum_{j=1}^{N} {}_{\ni\, j\neq i} A_{ij} \frac{r_j^{\frac{\omega_i}{\omega_j}}}{r_i} sin\ \omega_i \left(\frac{\theta_j}{\omega_j} - \frac{\theta_i}{\omega_i} + \frac{\emptyset_{ij}}{\omega_i\omega_j}\right) \quad (28)$$

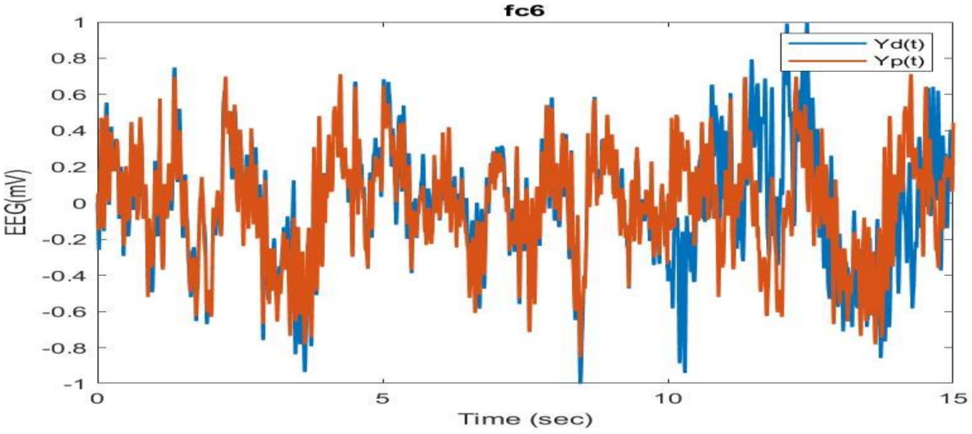

a

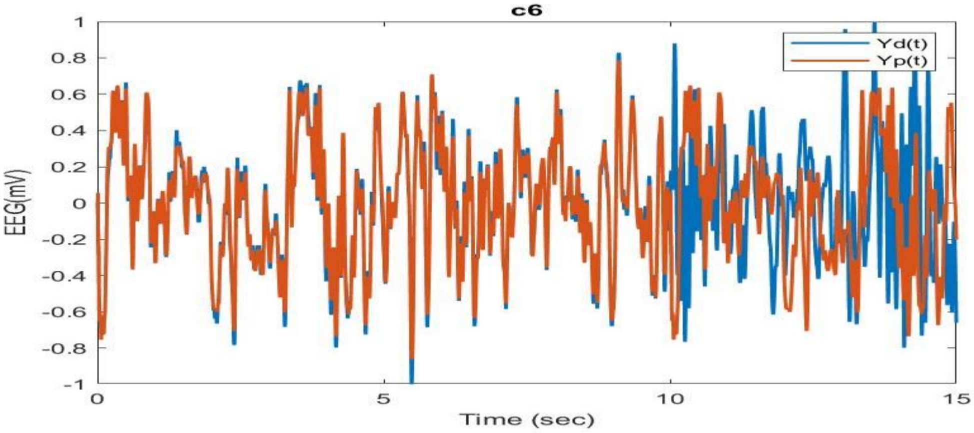

b

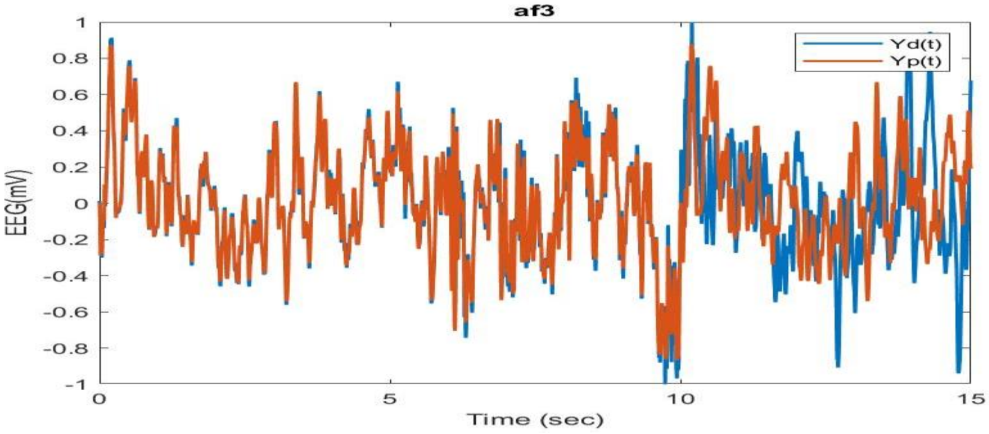

c

Supplementary Figure 7 (a-c): -Empirical EEG Data and training and testing (model reconstructed and model predicted) of five sleep stages (a: wake; b: NREM N2, c: REM). The blue line shows actual

EEG Data (Yd(t)) and orange line (Yp(t)) shows model reconstructed (initial 10 sec) and then predicted output (next 5 sec). Where initial 10 sec data are reconstructed and 5 sec data are predicted from model.

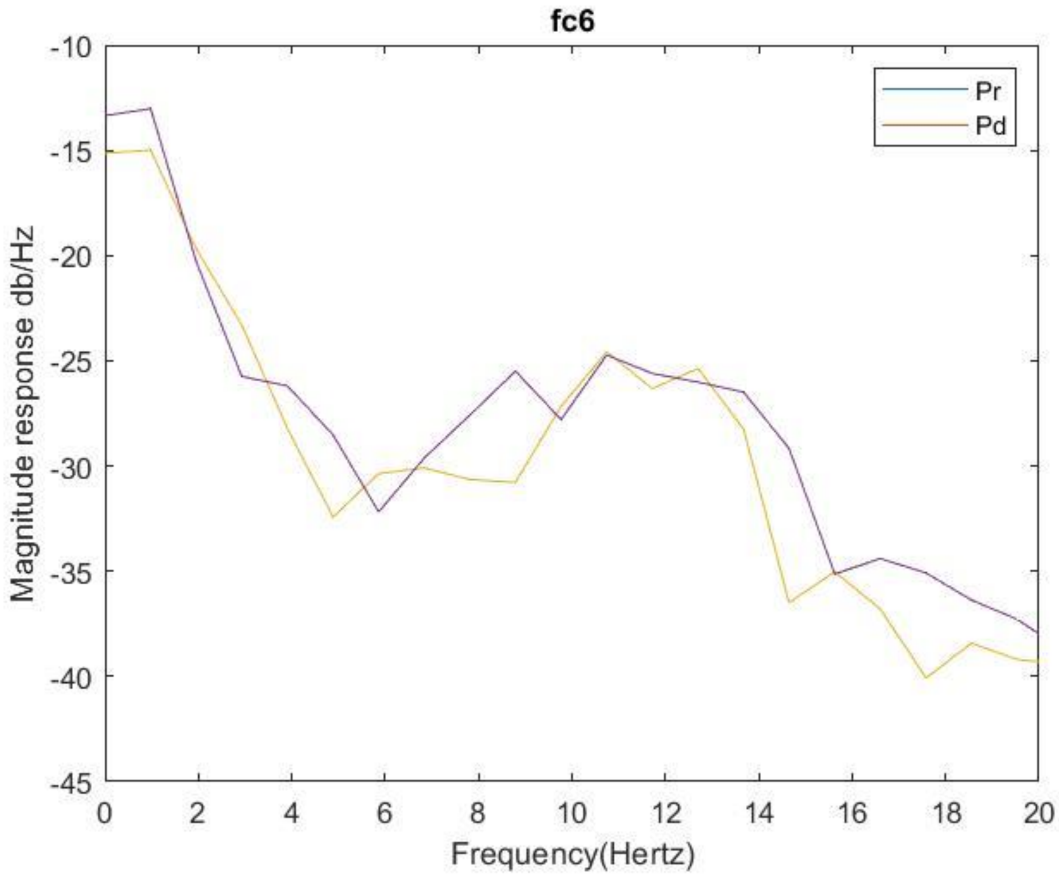

a

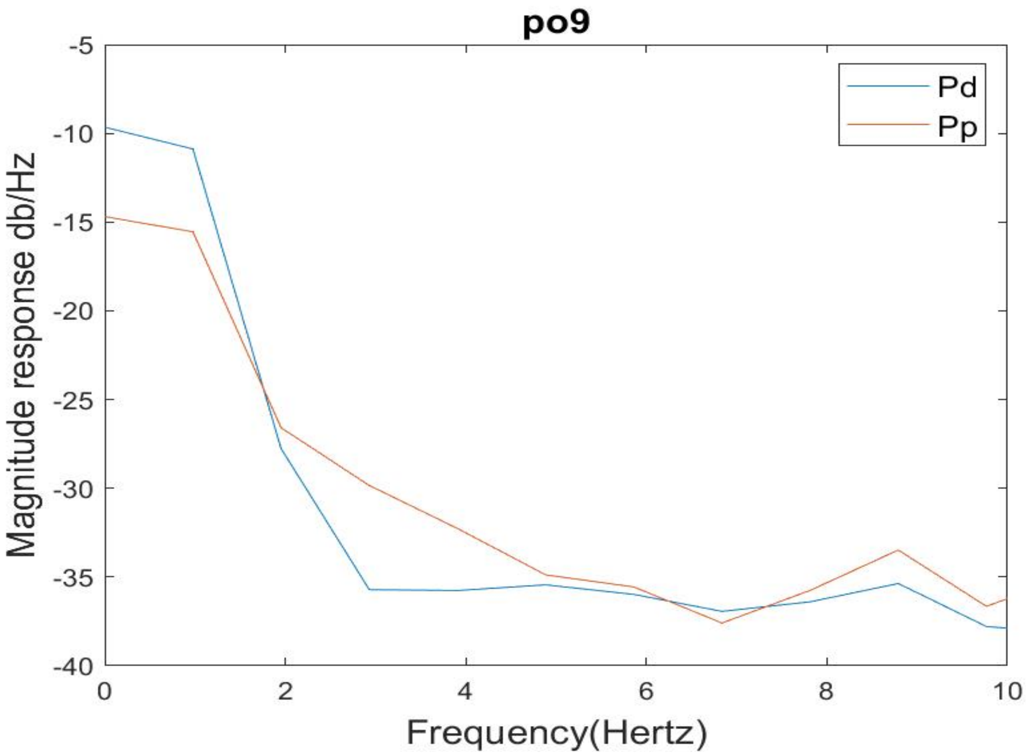
po9
Pd
Pp
Magnitude response db/Hz
Frequency(Hertz)

b

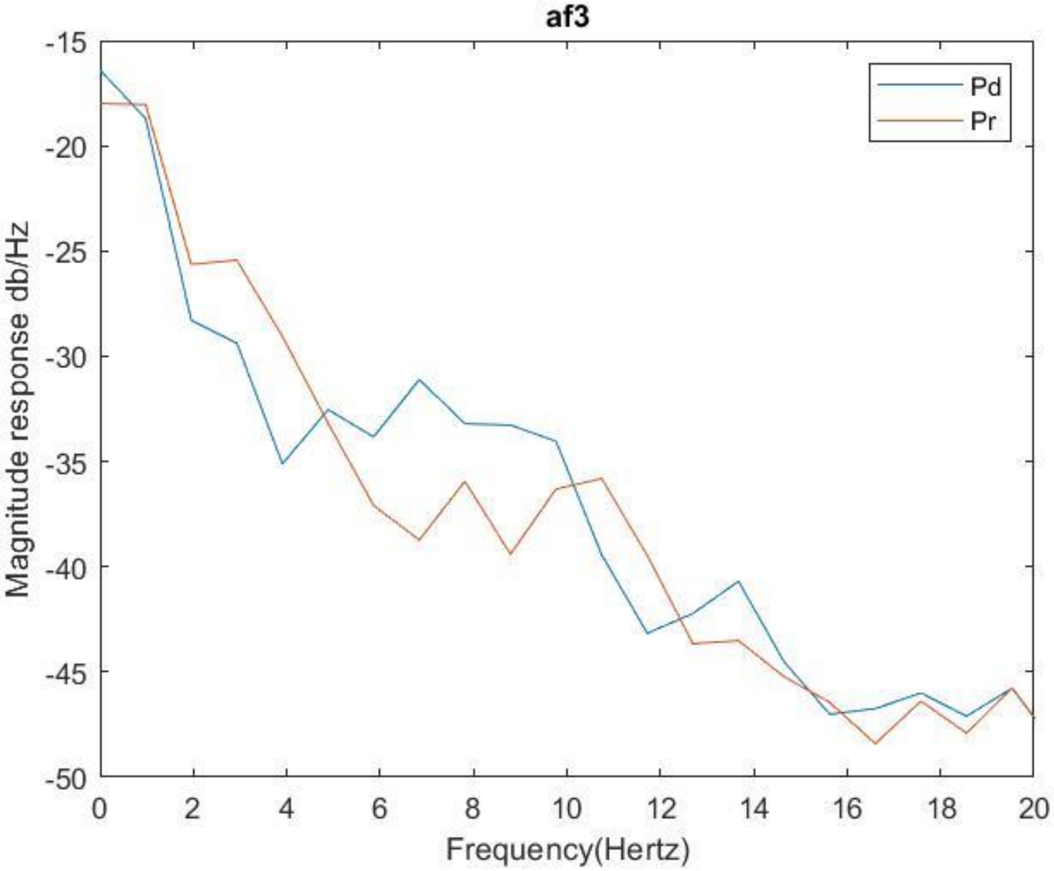
af3
Pd
Pr
Magnitude response db/Hz
Frequency(Hertz)

c

Supplementary Figure 8(a-c): Power spectral density curves of experimental signal and model predicted signal for a: wake; b: NREM N2, c: REM, the blue line shows power spectrum of actual EEG Data (Pd) and orange line (Pp) shows model predicted power spectrum.

10. **Spatial Distribution of Oscillators**

(i) Rectangular Grid:

Here we use 10 sec data of 8 electrodes from frontal and central lobes shown in fig 6(a-b). The signals from the 8 electrodes selected are modeled using the architecture described in section 2.8. Since the mapping between the oscillators and the electrodes is determined by the nearest neighbor criterion (eqn. 4a), it is expected that nearby electrodes are likely to share some oscillators. Training of the network proceeds as described below.

The network associated with each individual electrode - the hidden layer from which the electrode receives inputs, and the set of oscillators from which that hidden layer receives inputs – is trained separately (fig. 7). Here all 8 electrodes are selected, which form a typical rectangular grid, and the location of the electrodes in the Cartesian coordinate system is specified in Table (1). Training of the network associated with each electrode is done successively, from electrode to the next. Training begins with electrode C3 and covers all the electrode following the dotted arrows in fig. 3b.

Channel Name	X	Y	Z
C1	10	10	4
Cz	20	10	4
C2	30	10	4
Fc2	30	10	4
Fc1	10	10	4
F1	10	10	4
Fz	20	20	4

F2	30	20	4

Table 1: Location of 8 channel electrodes

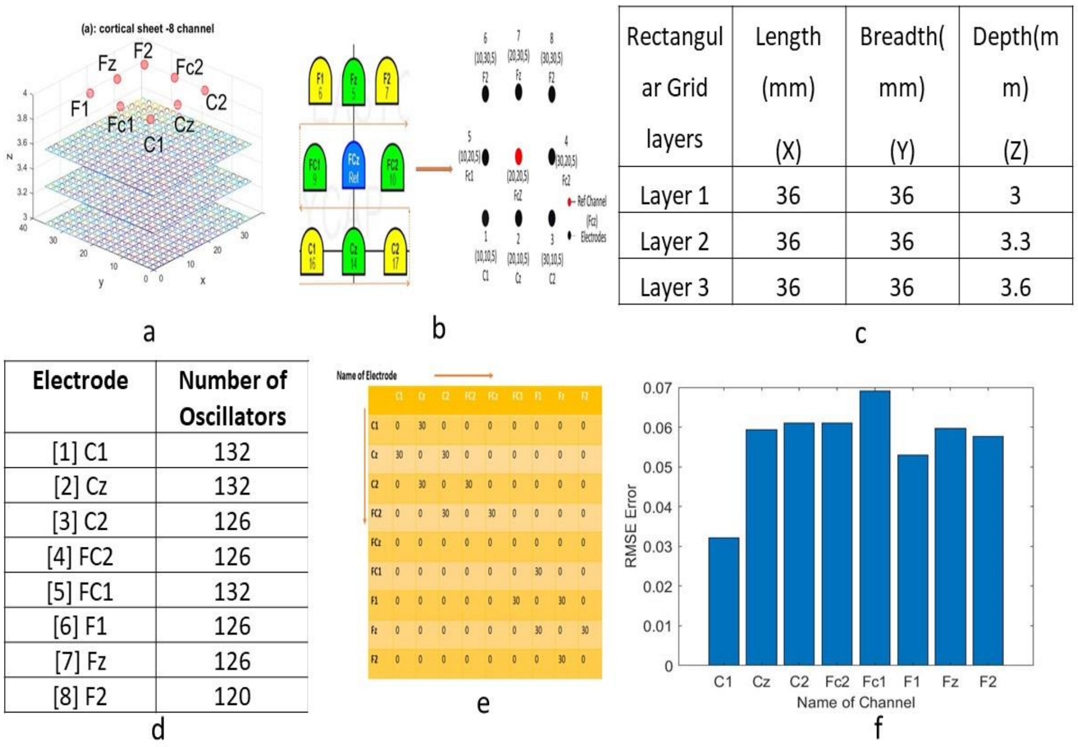

Supplementary Figure 9: a)3-layer cortical surface; b) Spatial map of Electrodes; c) Dimension of Rectangular Grid (d) The number of oscillators allocated to each channel; e) shared oscillators among the pair of channel; f) RMSE between desired and predicted signal presented for 8 channels in case of cortical layer with rectangular slab geometry.

We create a three-layer cortical surface with a rectangular geometry of dimensions (Fig. 3a and 3c). Each layer consists of 18 X 18 = 324 columns, and each column contains one oscillator. Hence totally 18 X 18 X 3 = 972 oscillators are present in the three layers. The layer of electrodes is placed on top of the 3 layered cortical surface. Next, we calculate the number of oscillators allocated to each electrode using eqn. (6a) with a distance threshold ($\xi_1 = 7.5$). All the oscillators in the cortical layer, whose distance from the individual electrode is less than (ξ_1), are allocated to that particular electrode. The number of oscillators allotted to various electrode are shown in Fig 3 d. Also, there is a separate hidden layer (consisting of 15 sigmoidal neurons) associated with each electrode. Note that channels that are close to each other in the electrode layer are likely to have more shared oscillators. Fig. 3 (e) provides the information about the number of shared oscillators among pairs of electrodes.

There is another threshold distance among oscillators ($\xi_2 = 1$), which determines the lateral connectivity among the oscillators using eqn. (4b). Only oscillator pairs whose mutual distance is less than ξ_2 are connected. The network thus designed, - with the oscillators connected among themselves over a neighborhood, and connected to the channels within a distance, with a separate hidden layer allotted to each electrode, - is trained on 8 channel EEG data.

11. Hurst Component

R/S method has been applied to estimate the Hurst exponent of a time series, which measures the long-range correlation. This measure has been broadly used in clinical EEG studies. Steps for the calculation of Hurst components are adopted from [3].

Finally, the Hurst exponent is calculated on the rescaled range [24] analysis on the time window of 2500 data points corresponding to next 5 sec. We have calculated the mean error and standard deviation of the difference between the model predicted signal's Hurst component and the experimental EEG signal's Hurst component. We found with our proposed network NREM N2 has the smallest error (Table 2)..

A. Hurst component prediction mean error:

Sleep Stages	Mean hurst component error
Wake	0.072±0.0061
NREM N1	0.014+-0.0134
NREM N2	**0.0088±0.0108**
NREM N3	0.0102±0.0075
REM	0.033±0.032

Table 2: Hurst component between predicted data and actual EEG for 5 sleep stages between model predicted and empirical EEG. The results were obtained by averaging over 56 channels for each sleep stage.

Error bar plot of Hurst exponent between our proposed model and Nagual et al.

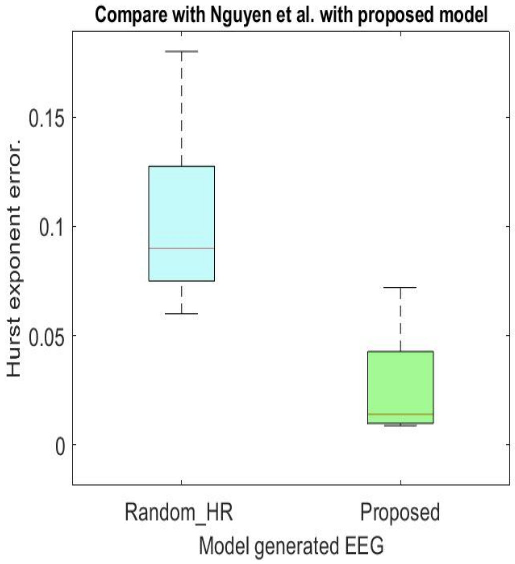

A

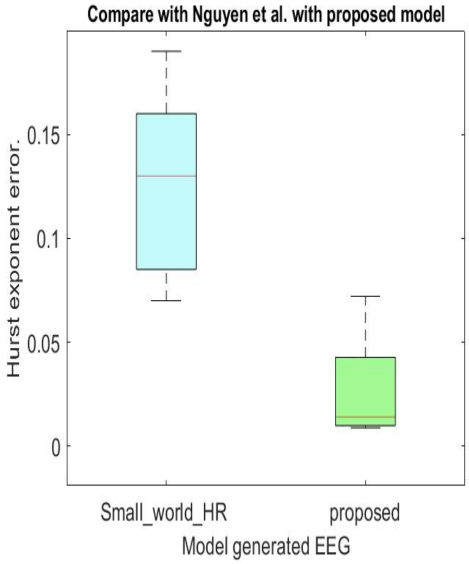

B

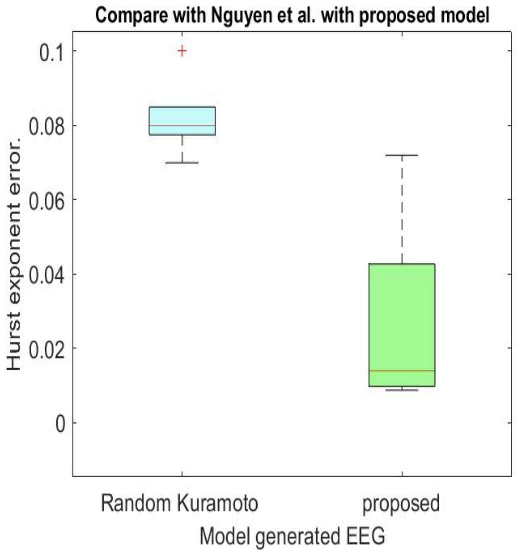

C

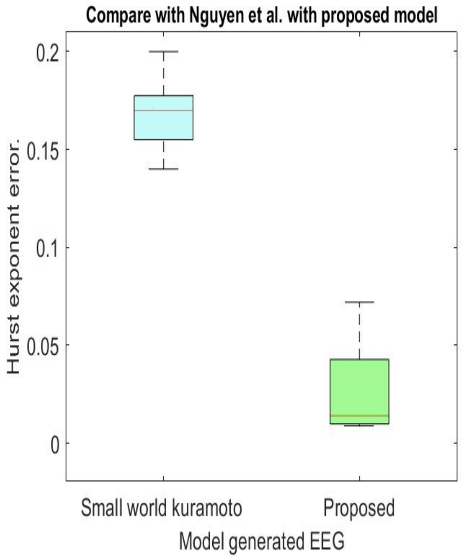

D

Supplementary Figure 10: EEG Hurst exponent distribution during prediction proposed by Nguyen et al (left bar in each figure), and our proposed coupled hopf network (right bar plot in each figure). Bar

plot is calculated using hurst component (HE) prediction error for all 5 set of EEG((i)-Wake, (ii)-NREM N1, (iii)-NREM N2, (iv)-NREM N3, (v)-REM) for 4 different network proposed by Nguyen et al. Similarly, HE prediction calculated from our proposed model for all 5 sleep stages (7. A-comparison between Random HR and Proposed; 7.B--comparison between small world HR and Proposed; 7.C- -comparison between Random Kuramoto and Proposed; 7.D--comparison between small world Kuramoto and Proposed).

12. Higuchi fractal dimension (HFD)

We have compared the signal complexity in terms of Higuchi fractal dimension (HFD) [4] which is calculated from model predicted (simulated) and empirical EEG Data. The HFD was calculated from model predicted dataset during wake (mean: 1.1561, SD: 0.1104) and NREM N1(mean: 1.0647, SD: 0.1405). Similarly, the HFD values are calculated from empirical datasets during wake (mean: 1.2085, SD: 0.1301) and NREM N1 (mean: 1.1594, SD: 0.0913). The goal of this analysis to compare HFD of model predicted wake with the HFD of model predicted NREM N1. To this end, ANOVA was conducted and the results ($F: 12.19$, $p< 0.02$) showed that complexity was significantly higher in Wake. A similar study was conducted with empirical data showing the same data trend ($F=6.93$, $p<0.02$). By using this following statistical analysis, we can say that the model shows a similar behaviour like actual EEG.

13. Statistical Test on Power spectrum density

Univariate statistical t-test was done to identify the relative significance of power spectral density between the EEG signal predicted by the model and empirical EEG Data.

Sleep stages	Predicted power spectrum(avg.)	Predicted power spectrum(std)	Empirical power spectrum(avg)	Empirical power spectrum(std)	p-value
Wake	43.1617	33.7570	50.5943	43.1744	0.137
NREM n1	45.704	40.82	49.34	35.61	0.376
NREM n2	44.10	33.13	54.67	23.68	0.144
NREM n3	42.86	43.98	43.98	56.001	0.615
REM	37.66	30.23	53.69	37.07	0.0003

Table 3: Statistical significance(p-values) of power spectral densities when comparing model predicted EEG with actual EEG

According to the result presented in the above table (table 2) Wake, NREM N1, NREM N2, NREM N3 are not statistically significant. Hence in these 4 stages of sleep model prediction in terms of power spectral density is more accurate and matches well with the experimental EEG's power spectrum. However, in case of REM stage the difference in power spectrum density is statistically significant (p value < 0.05).

14. Spherical shell model:

Cartesian coordinate system:

**Channel Name**	**X(mm)**	**Y(mm)**	**Z(mm)**
F1	60.738	0	59.463
FZ	59.913	-26.042	54.381
F2	59.874	-26.025	54.431

Table 4: Sample location in Cartesian coordinate system from EEGLAB

**Channel Name**	**Total Oscillators**
F1	116
Fz	132
F2	129
FC2	186
FC1	177
C1	113
Cz	219

C2	149

Table 5: The number of oscillators allotted to each elctrode

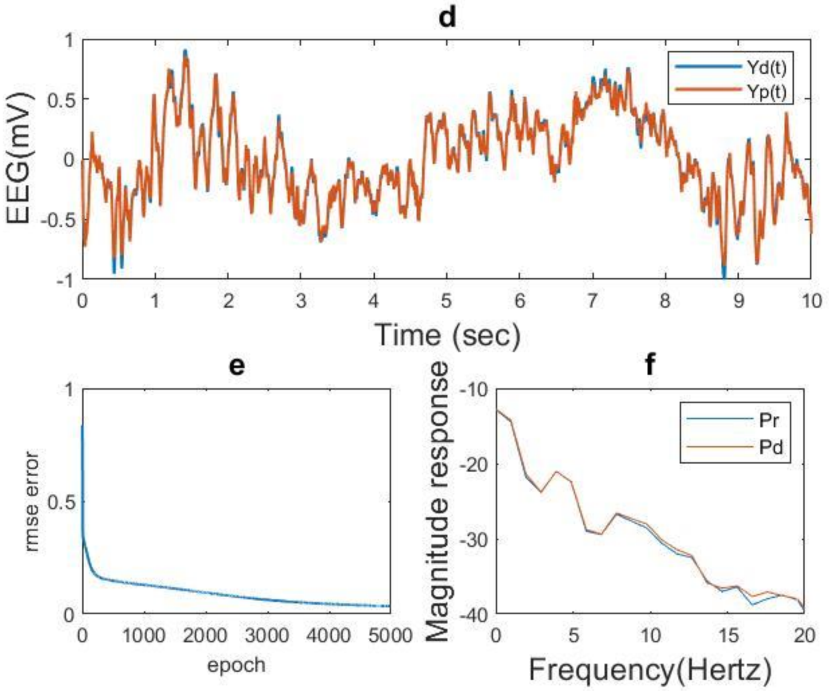

Supplementary Figure 11 (d-f): Time series of reconstructed signal and the desired signal for C4 channel among 8 channels in a spherical shell, during training stage. (e)- RMSE error w.r.t. training epochs, (f)-power spectrum of desired and reconstructed signal, $Y_d(t)$ =desired EEG, $Y_p(t)$=predicted EEG, P_d=desired EEG power.

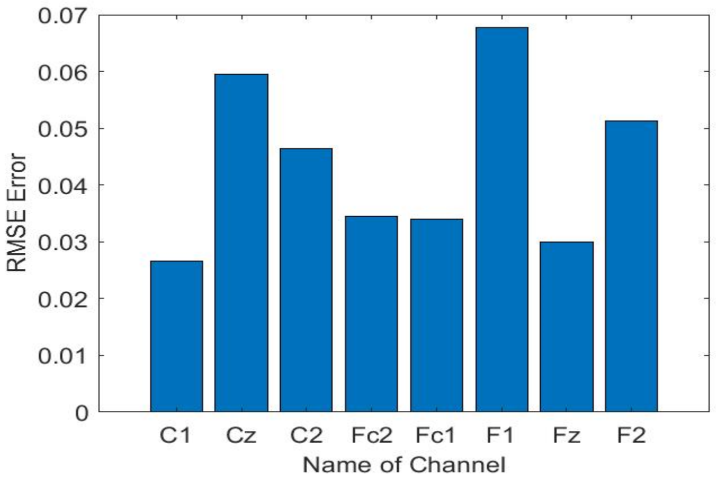

Supplementary Figure 12: RMSE between desired and reconstructed during training signals: 8 channels and Spherical shell geometry of the cortical layer.

15. Detail description on BONN Dataset

This dataset was recorded from the Department of Epileptology at the University of Bonn, Germany [10]. There are 5 sets of EEG data (A-E), containing 100 single channel EEGs of duration 23.6 sec, recorded at 173.61 Hz sampling frequency. The recorded EEG data are preprocessed by bandpass filtering (0.53 to 70 Hz). There are 4097 samples in each dataset. Sets A and B were collected on five healthy volunteers, who were relaxed in an awake state with eyes open (A) and eyes closed (B), respectively. From five patients, sets C and D were recorded during the seizure-free interval, and set E was recorded during seizure occurrence. In this present study, we have done six different combinations of classification studies.

16. Comparison with Bandyopadhyay et al. [9]

Supplementary Table 5: comparison with Bandyopadhyay et al [9]

	Bandyopadhyay et al[9]	Proposed work
Type of model	Hopf oscillator with hidden layer and two-stage training	Hopf oscillator with hidden layer and two-stage training
Signal used in the model	fMRI with 160 ROI	Sleep EEG of different sleep stages-62 channels

Lateral connection/Method	Structural connectivity was used.	No such structural connectivity was used.<br><br>Here based on the spherical/rectangular model, Individual electrodes have a bunch of oscillators, and nearby two electrodes have a few common oscillators also, their learned frequency and phase have been used. We introduce spherical geometry based on 10-20 electrode coordinate system extracted from EEGLab.
Main Result	The effect of information loss due to structural connectivity damage due to some disease conditions has been shown	Future prediction of the signal has been shown after training. And all the properties (time series, Hurst component, and power spectrum) have been explored.<br><br>More interestingly this work shows the predicted signal (during testing) follows real EEG signal (for different sleep stages). See statistical test.
Analysis matrix	Correlation coefficient between empirical and simulated signals has been calculated	1. MAE error<br>2. Power spectrum error<br>3. Hurst component error<br><br>between empirical and simulated signal has been calculated
key Findings	The effect of loss of structural information on functional information due to dieseases condition in terms of correlation coefcient estimated on simulated and empirical FCs.	Can be useful fo synthetic EEG generation. The predicted signal has good agreement with actual EEG with respect to power spectrum, Hurst exponent, Sensitivity analysis- We have shown at which value of tuning parameters (μ, ζw, $\boldsymbol{\beta}$), the network performs optimally.

Supplementary References:

[1] Georgiou, G. M., & Koutsougeras, C. (1992). Complex domain backpropagation. *IEEE transactions on Circuits and systems II: analog and digital signal processing*, *39*(5), 330-334.

[2] Aydore, S., Pantazis, D., & Leahy, R. M. (2013). A note on the phase locking value and its properties. *Neuroimage*, *74*, 231-244.

[3] Rahmani, B., Wong, C. K., Norouzzadeh, P., Bodurka, J., & McKinney, B. (2018). Dynamical Hurst analysis identifies EEG channel differences between PTSD and healthy controls. *PloS one*, *13*(7), e0199144.

[4] Vega, C. F., & Noel, J. (2015, June). Parameters analyzed of Higuchi's fractal dimension for EEG brain signals. In *2015 Signal Processing Symposium (SPSympo)* (pp. 1-5). IEEE.

[5] Rubinov, M., & Sporns, O. (2010). Complex network measures of brain connectivity: uses and interpretations. *Neuroimage*, *52*(3), 1059-1069.

[6] Noble, S., Scheinost, D., & Constable, R. T. (2019). A decade of test-retest reliability of functional connectivity: A systematic review and meta-analysis. *Neuroimage*, *203*, 116157.

[7] Iravani, B., Arshamian, A., Fransson, P., & Kaboodvand, N. (2021). Whole-brain modelling of resting state fMRI differentiates ADHD subtypes and facilitates stratified neuro-stimulation therapy. *Neuroimage*, *231*, 117844.

[8] Cofré, R., Herzog, R., Mediano, P. A., Piccinini, J., Rosas, F. E., Sanz Perl, Y., & Tagliazucchi, E. (2020). Whole-brain models to explore altered states of consciousness from the bottom up. *Brain Sciences*, *10*(9), 626.

[9] Bandyopadhyay, A., Ghosh, S., Biswas, D., Chakravarthy, V. S., & S. Bapi, R. (2023). A phenomenological model of whole brain dynamics using a network of neural oscillators with power-coupling. *Scientific Reports*, *13*(1), 16935.

[10] Andrzejak, R. G., Lehnertz, K., Mormann, F., Rieke, C., David, P., & Elger, C. E. (2001). Indications of nonlinear deterministic and finite-dimensional structures in time series of brain electrical activity: Dependence on recording region and brain state. *Physical Review E*, *64*(6), 061907.
